# Efficacy of surgical methods for peri-implantitis: a systematic review and network meta-analysis

**DOI:** 10.1186/s12903-023-02956-6

**Published:** 2023-04-19

**Authors:** Jing Cheng, Liang Chen, Xian Tao, Xiang Qiang, Ruiying Li, Jia Ma, Dong Shi, Zijin Qiu

**Affiliations:** 1 Stomatological Hospital of Xiamen Medical College, Xiamen Medical College, Xiamen, People’s Republic of China; 2 Xiamen Key Laboratory of Stomatological Disease Diagnosis and Treatment, Xiamen, People’s Republic of China; 3 Department of Periodontology, Peking University School and Hospital of Stomatology & National Center for Stomatology & National Clinical Research Center for Oral Diseases & National Engineering Laboratory for Digital and Material Technology of Stomatology, Beijing, People’s Republic of China

**Keywords:** Peri-implantitis, Dental implant, Surgical treatment

## Abstract

**Background:**

Peri-implantitis is the most difficult biological complication associated with dental implants, often requiring surgical treatments in advanced stages. This study compares the effectiveness of different surgical methods for peri-implantitis.

**Methods:**

Randomized controlled trials (RCTs) of different surgical treatments for peri-implantitis were extracted from EMBASE, Web of Science, Cochrane Library databases, and PubMed systematically. Pairwise comparisons and network meta-analyses (NMA) were conducted to analyze the effect of surgical treatments on probing depth (PD), radiographic bone fill (RBF), mucosal recession (MR), bleeding on probing (BOP), and clinical attachment level (CAL). In addition, risk of bias, quality of evidence, and statistical heterogeneity of the selected studies were evaluated. A total of 13 articles were included in this study, involving open flap debridement (OFD), resective therapy (RT), and augmentative therapy (AT) with and without adjunctive treatments (laser therapy, photodynamic therapy, local antibiotics, phosphoric acid, and ozone therapy).

**Results:**

AT improved RBF and CAL more than OFD, but does not outperform OFD in reducing peri-implant soft-tissue inflammation. AT, OFD and RT did not significantly alter the levels of MR. Addition of ozone therapy improved the effect of AT, but addition of photodynamic therapy did not affect PD reduction and CAL gain significantly. Similarly, adjuvant treatment with phosphoric acid during RT did not significantly change the outcome of BOP.

**Conclusions:**

Within the limitation of this systematic review and NMA, AT was superior to OFD in improving peri-implantitis outcomes. While adjunct use of ozone therapy may further improve the efficacy of AT, the limited evidence supporting this combination therapy argues for cautionary interpretation of these results.

**Supplementary Information:**

The online version contains supplementary material available at 10.1186/s12903-023-02956-6.

## Background

Over the past few decades, dental implants have been extensively used as a treatment alternative to conventional removable partial-fixed dentures. The long-term survival rate of the implants ranges from 92.3 to 95.7% [1, 2]. With the popularization of dental implant therapy, however, various implant complications have emerged, including biomechanical overload, infection or inflammation, and other issues [3]. One of the main complications of implantation is peri-implantitis, which exhibits a nonlinear and accelerated pattern of bone loss and may ultimately result in implant loss [4]. Peri-implantitis is defined as “a pathological condition occurring in tissues around dental implants, characterized by inflammation in the peri-implant mucosa and progressive loss of supporting bone” [5]. Other typical characteristics of peri-implantitis include bleeding on probing and/or suppuration, increased probing depths and/or recession of the mucosal margin [6]. The prevalence of peri-implantitis ranges from 11.2 to 22% [7–9], with risk factors such as history of periodontitis, smoking, diabetes, poor plaque control and lack of regular maintenance therapy [6]. Nowadays, peri-implantitis is considered to be the most difficult biological complication associated with implants, as untreated disease can eventually lead to implant loss [10].

Treatment options for peri-implantitis comprise non-surgical and surgical therapy but there is no reliable evidence to suggest which interventions are most effective [11]. The nonsurgical method is effective in reducing soft tissue inflammation such as bleeding on probing (BOP) [12], but the efficacy of treatment is limited [13, 14]. Surgery is often recommended for advanced peri-implantitis [10, 15] and can be divided into three categories: access flap surgery; resective therapy; augmentative methods, which can also be combined with other treatment modalities [16]. The access flap method, i.e., open flap debridement, mitigates inflammation around the implant by exposing the implant surface and applying debridement directly [17, 18]. Resective therapy is indicated for supracrestal bone defects with threads exposed in esthetically non-demanding areas. The procedure involves reduction or removal of pathological peri-implant pockets, apical mucosal flap placement, or bone recontouring with or without implant surface modification, known as implantoplasty [19]. Augmentative methods are also known as regenerative treatment, and involve flap elevation, mechanical debridement, and placement of graft material with or without membrane [20]. This surgical method aims to regenerate bone defects, achieve re-osseointegration, and limit peri-implant soft-tissue recession [21]. Often, the above three surgical treatments are combined with adjuvant therapy such as laser therapy, photodynamic therapy, and local antibiotics.

Surgical treatments of peri-implantitis are shown to have better outcomes than non-surgical treatments [22]. Chan et al. showed that surgical treatments result in an estimated probing depth (PD) reduction of 2–3 mm, and regenerative procedures can achieve an average radiographic bone filling of 2 mm [23]. Moreover, it is more effective to use bone graft materials in combination with barrier membranes. Although partial filling of defects may be expected, complete filling of the bone defect caused by peri-implantitis using the guided bone regeneration (GBR) protocol is unpredictable [24]. Cases of implant loss, disease recurrence, and further progression have been reported even though augmentative therapies were clinically and radiographically successful [25]. Despite promising results that have been achieved in regenerative treatments, non-regenerative modalities have limited effects. Given the many therapeutic options available for peri-implantitis, evaluating the clinical effects of the different surgical treatments is essential for clinical decision making. Conventional meta-analyses on the surgical treatment of peri-implantitis typically use pairwise comparisons which do not simultaneously evaluate the many surgical methods available [26]. Network meta-analysis can combine the effects of multiple treatments and make statistical comparisons [14]. To the best of the author’s knowledge, there is a dearth of network meta-analysis that evaluates the efficacy of access flap surgery, resective therapy, and augmentative methods, both with and without adjunctive treatments.

Thus, this systematic review and network analysis aim to screen recent research on surgical treatment methods for peri-implantitis. In this study, the efficacy of various surgical treatment modalities was evaluated in accordance with the resolution of different clinical and radiographic parameters. This review endeavors to provide a reference for clinicians to select the most appropriate surgical treatment method.

## Methods

### Protocol registration and report format

Protocol for the present review was registered with the identification number CRD42022313804 in the PROSPERO database, hosted by the National Institute for Health Research, University of York, Center for Reviews and Dissemination. Our manuscript was prepared based on the Cochrane Collaboration guidelines [27], and the data reported in accordance with the Preferred Reporting Items for Systematic Reviews and Meta-Analysis Extension Statement for systematic reviews incorporating network meta-analysis for healthcare interventions [28].

### Objectives

To evaluate the clinical effect of surgical methods on periodontal parameters in patients with peri-implantitis.

### PICOS questions

- Population (P): systemically healthy adult patients with peri-implantitis.
- Intervention (I): surgical therapies for peri-implantitis, including access flap surgery, resective therapy, augmentative therapy with adjunctive methods such as laser, photodynamic therapy, and so on.
- Comparator (C): open flap debridement alone applied for peri-implantitis.
- Outcomes (O): 1) probing depth (PD) reduction (in millimeters): changes in distance between the gingival margin and the bottom of the probeable pocket before and after treatment with positive values indicating decreased PD after the intervention; 2) radiographic bone fill (RBF; in millimeters): radiographic assessment of bone gain after the intervention; 3) mucosal recession (MR; in millimeters): buccal recession at the peri-implant mucosal margin after the intervention; 4) bleeding on probing (BOP) reduction (percentage): change in percentage of sites with bleeding on probing; 5) clinical attachment level (CAL) gain (in millimeters): changes in distance between the implant neck and the bottom of probeable pocket before and after treatment with positive values indicating increased CAL after the intervention.
- Study (S): Randomized controlled trials (RCTs) only.

### Inclusion criteria

- 1) RCTs of systemically healthy patients with peri-implantitis.
- 2) Involved surgical methods for peri-implantitis therapy.
- 3) Reported at least one clinical or radiographical parameter.
- 4) Patients were followed up for at least 3 months after surgical intervention.
- 5) Screw-shaped implants with either smooth or rough surfaces were included.

### Excluded criteria

- 1) Pre-clinical articles, animal studies, reviews, and case reports.
- 2) Reports with duplicated data.
- 3) Insufficient/unclear data.
- 4) Lack of clinical data on changes in PD, CAL, BOP, RBF or MR.

### Information sources and literature search

Four electronic databases (EMBASE, Web of Science, Cochrane Library databases, and PubMed) were searched between January 1, 2000 and May 28, 2022 for relevant articles on surgical treatment of peri-implantitis. Unpublished literature, gray literature, non-profit reports, government articles, or other materials were searched via the ClinicalTrial.gov website. The search strategy includes "peri-implantitis" AND "surgical procedure". Furthermore, a manual search was conducted in dental and implant-related journals, including the Journal of Dental Research, Journal of Clinical Periodontology, Journal of Periodontology, Clinical Oral Implants Research, Clinical Implant Dentistry, and Related Research, The International Journal of Oral & Maxillofacial Implants, Journal of Oral and Maxillofacial Surgery, International Journal of Oral Implantology, Clinical Oral Investigations, as well as International Journal of Periodontics and Restorative Dentistry. Moreover, references of the included articles were searched to identify publications that were not identified electronically.

### Selection of the articles

Titles and abstracts from the electronic search were first screened by two reviewers independently. Articles that were considered to be potentially relevant by at least one reviewer entered the next screening step. Full manuscripts were acquired if the title and summary satisfied the eligibility criteria, or if insufficient information is available. Any disagreement between the reviewers was resolved through discussion. Articles that did not satisfy the inclusion criteria were excluded with reasons provided.

### Data extraction

All pertinent information including first author, year of publication, follow-up, age of participants, intervention and control group, surgical method, as well as mean changes in periodontal parameters (PD, CAL, BOP, RBF, MR) ± SD were independently retrieved from the selected articles by two reviewers. At any stage, disagreements between the reviewers were resolved through open discussion and consensus. Unresolved disagreements were evaluated by a third reviewer to settled the discussion.

### Network meta-analysis

The frequentist method was adopted for network meta-analysis using the STATA software (version: 14.1, StataCorp LLC, TX, USA), R language (version: 4.1.3) [29], as well as the “netmeta” package (version: 2.1–0) [30, 31]. R was mainly used for data analysis, while the STATA software was used to optimize visual charts. All article reported data were used to obtain the total mean difference using a random effects model for total mean deviation, and pairwise comparison was drawn based on 95% confidence intervals. Global and local methods were employed for the network consistency algorithm [32]. Statistical heterogeneity was evaluated using τ^2^ and *I*^2^ statistics. There is low heterogeneity (*I*^2^ < 25%), medium heterogeneity (25–75%), and high heterogeneity (*I*^2^ > 75%) [33]. Publication biases were evaluated using the Comparison-adjusted funnel plot. Network plots were generated using the STATA software program. Based on previous meta-analyses [14, 34], changes in the respective parameters were expressed as mean difference (MD) with 95% confidence interval for the intuitive clinical interpretation of the results. League tables were generated using netmeta to show the effects of different surgical treatments on the soft and hard tissues around implants. Surface under the Cumulative Ranking Curve (SUCRA) was used to rank the included methods for each outcome and assess the probability of the intervention being the best option [35]. Treatments with higher SUCRA scores are more effective, while those with lower SUCRA scores are less effective.

### Evaluation of risk of bias and overall quality of the evidence

Risk of bias of the included trials was assessed by the Cochrane Risk of Bias Assessment tool (ROB 2) and include five domains: bias arising from the randomization process, bias due to deviations from intended interventions, bias due to missing outcome data, bias in measurement of the outcome, and bias in selection of the reported result [36]. Trials were classified as low risk only if all five domains scored low, whereas trials with high risk in one or more domains were considered to have high risk of bias. Credibility of the findings from each network meta-analysis was evaluated using the Confidence in Network Meta-Analysis (CINeMA) online documentation (http://cinema.ispm.ch/#doc) [37, 38], which classified the risk of bias for each study into low risk, unclear risk, or high risk of systematic errors. All evaluations were performed independently by three reviewers in triplicates. Any disagreements were resolved by public discussion until a conclusion was drawn.

## Results

### Study selection

**Fig. 1:**
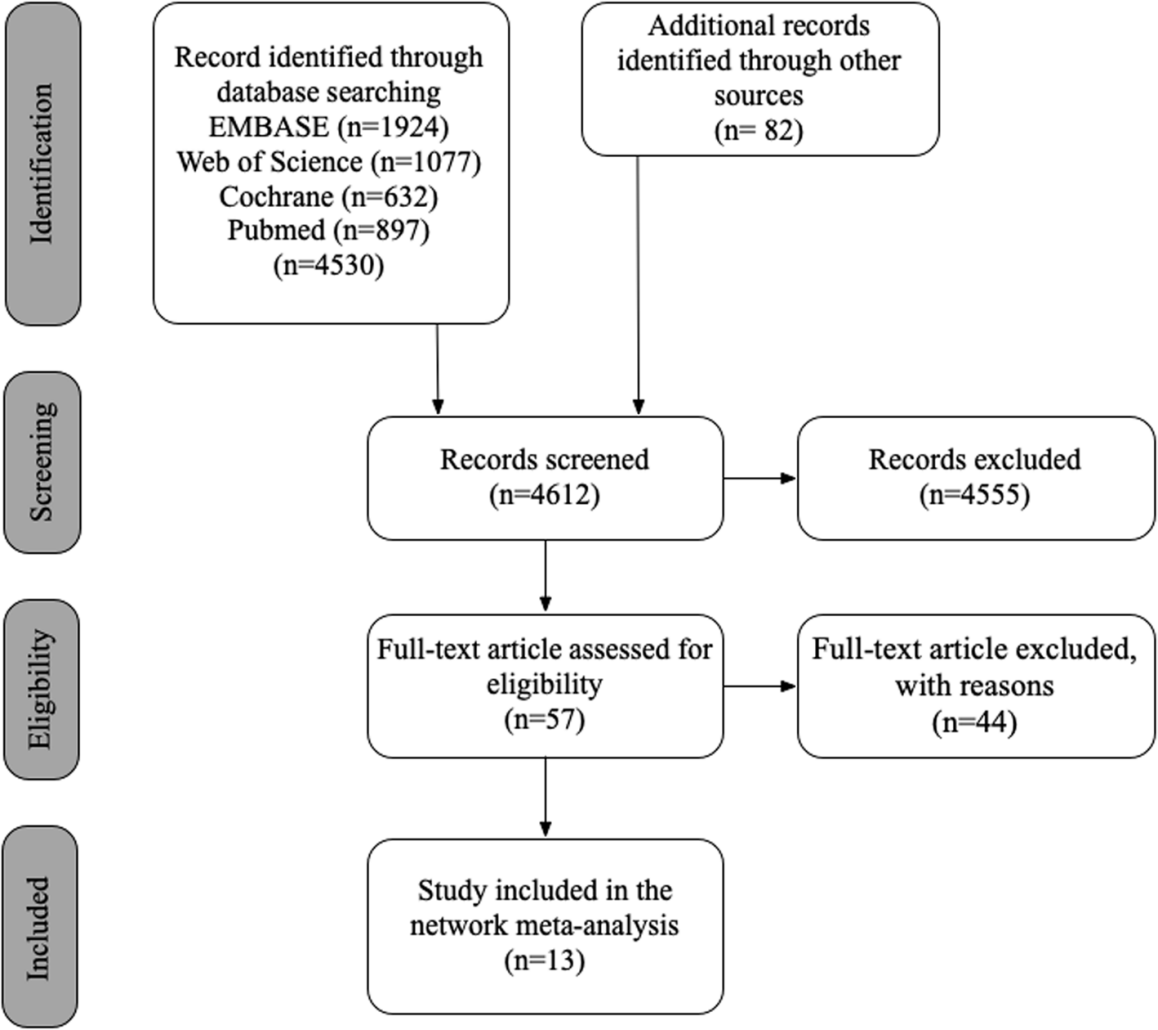
Schema outlining the search process

Figure 1 illustrates the detailed process of study selection. Four electronic databases and a manual search of relevant articles yielded 4612 articles, 57 of which were selected for full text evaluation. Excluded studies and the major reasons for their exclusion are described in the supplementary material (Appendix 5). A total of 13 articles were included in the network meta-analysis. The Kappa values for the consistency between the reviewers in terms of title/abstract screening and full-text evaluation reached 0.92 and 0.90, respectively, corresponding to an "almost perfect" consistency among the reviewers.

### Description of articles

Characteristics of the 13 selected articles are presented in Table 1. Randomized clinical trials (RCTs) with parallel group designs were used in all articles. All articles were published between 2012 and 2021 and the follow-up time ranged from three to 12 months. The studies included patients with an average age range of 46 to 73.5 years and received at least one implant. Periodontal parameters, including probing depth (PD), clinical attachment level (CAL), plaque index (PI), bleeding on probing (BOP), gingival index (GI), mucosal recession (MR), suppuration (SUP), and vertical defect depth (VDD), were adopted to express changes around the implants after the treatment. Five articles compared treatment efficacy between open flap debridement (OFD) and augmentative therapy (AT) [39–43], and one article compared OFD and OFD with local antibiotics (LA) [44]. Two articles compared OFD and OFD with photodynamic therapy (PDT) [45, 46]. One article compared resective therapy (RT) and RT with phosphoric acid (PA) [47]. One article compared AT and AT + PDT [48]. One article compared AT and AT with ozone therapy (AT + O) [49]. Finally, two articles compared RT and OFD [50, 51].

**Table 1:** Characteristics of the included articles and their interventions

Publication	Country	Follow-up times (months)	Participant age (years)	Control group	Test group	Number of intervention and comparison	Outcomes
Renvert S (2021) [39]	Sweden, France, Germany	12	CG: 62.9 ± 13.0 TG: 62.2 ± 10.2	OFD	AT	32/34	BL/PD/BI/SUP/REC
Cha JK (2019) [44]	South Korea	6	61.6 ± 21.6	OFD	OFD + LA	25/25	PD/PI/GI
Renvert S (2018) [40]	Sweden	12	CG: 70 ± 7.8 TG: 67.5 ± 11.3	OFD	AT	20/21	PD/BL
Isler SC (2018) [49]	Turkey	12	CG: 54.18 ± 10.36 TG: 54.4 ± 8.08	AT	AT + O	21/20	PI/GI/BOP/PD/CAL/REC/VDD
Albaker AM (2018) [45]	Saudi Arabia	12	CG: 61.5 ± 9.9 TG: 58.4 ± 8.0	OFD	OFD + PDT	13/11	PI/BOP/PD/BL
Hentenaar DFM (2017) [47]	Netherlands	3	CG: 57.0 ± 13.7 TG: 60.9 ± 7.2	RT	RT + PA	20/30	BOP/SUP/PD
Rakašević D (2016) [48]	Serbia	3	CG: 60 TG: 57.59	AT	AT + PDT	19/21	PD/CAL/BOP
Jepsen K (2016) [41]	Germany	12	58.4 ± 12.3	OFD	AT	26/33	VDD/PD/BOP/PI
Hamzacebi B (2015) [42]	Turkey	6	60.98 ± 11.9	OFD	AT	19/19	BOP/PD/REC/CAL/KM
Bombeccari GP (2013) [46]	Italy	6	46 ± 13.0	OFD	OFD + PDT	20/20	PD/CAL/BOP
Wohlfahrt JC (2012) [50]	Norway	12	CG: 65.0 ± 10.0 TG: 57.2 ± 12.3	OFD	RT	16/16	PD/BOP
Emanuel N (2012) [43]	Israel	12	64.81 ± 7.61	OFD	AT	14/18	PD/CAL/BL/BOP/REC
Lasserre JF (2020) [51]	Belgium	6	66.5 ± 24.5	RT	OFD	15/14	PI/BOP/SOP/PD/CAL/REC/BL

*CG* control group, *TG* test group, *OFD* open flap debridement, *AT* augmentative therapy, *RT* resective therapy, *OFD* + *LA* open flap debridement combined with local antibiotics, *OFD* + *PDT* open flap debridement combined with photodynamic therapy, *RT* + *PA* resective therapy combined with phosphoric acid, *AT* + *PDT* augmentative therapy combined with photodynamic therapy, *AT* + *O* augmentative therapy combined with ozone therapy, *PD* probing depth, *CAL* clinical attachment level, *PI* plaque index, *BOP* bleeding on probing, *GI* gingival index, *MR* mucosal recession, *SUP* suppuration, *VDD* vertical defect depth, *BL* bone level, *BI* bleeding index, *KM* keratinized mucosa, *SOP* suppuration on probing

### Risk of bias and quality of evidence

**Fig. 2:**
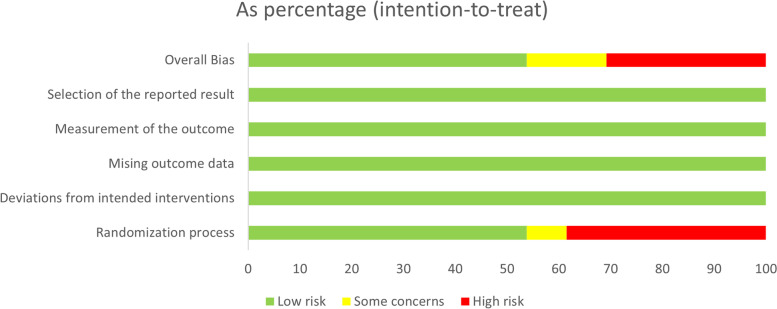
Graph showing the risk of bias categories and the percentage of articles with these risks

The risk of bias was assessed for 36 outcomes from the included randomized controlled trials. In the randomization process, 16 (44.4%) outcomes were at high risk of bias, 2 (5.5%) contained some concerns and the others were at low risk of bias. All outcomes were at low risk of bias in the other four domains (Fig. 2). The overall risk of bias was low in 7/13 (53.0%) trials assessing PD, 5/8 (62.5%) trials assessing RBF, 1/5 (20%) trials assessing MR, 4/8 (50%) trials assessing BOP and 2/5 (20%) trials assessing CAL (Appendix 12). A comprehensive examination of the comparison-adjusted funnel plots revealed the absence of any significant asymmetry, suggesting the absence of significant publication bias among the studies that were included in the analysis (Appendix 8). The overall quality of evidence in the pair-wise meta-analysis was graded using CINeMA (see Appendix 13 for details). The high risk is mainly concentrated in three domains: within-study bias, imprecision, and incoherence. Overall, the evidence of the included articles is very weak.

### Synthesis of results from the network meta-analysis

**Fig. 3:**
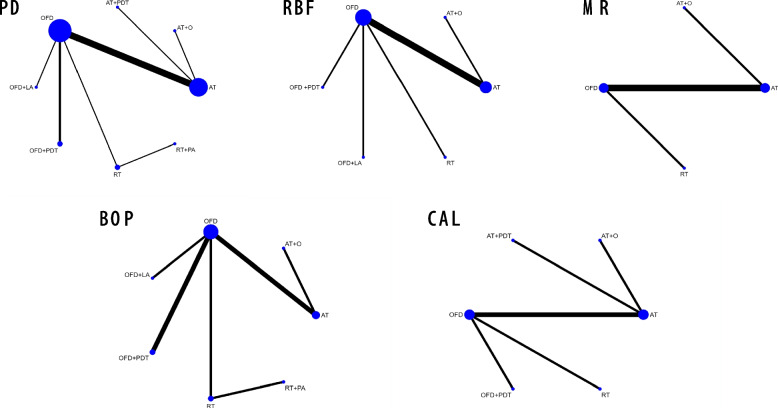
Node link diagram showing the network meta-analysis comparisons. Nodes represent treatment methods, lines represent comparisons between the linked nodes, size of node and lines represent number of articles involved in that comparison

The network meta-analysis (NMA) of the 13 articles is presented as node link diagrams (Fig. 3) where the 8 nodes represent the 8 treatment methods, the line between nodes represents direct comparison, and the size of nodes and the thickness of the lines represent the number of articles, i.e. bigger nodes and thicker lines contain more articles in the comparison between those methods. The network analysis of PD involved a total of 13 studies, which comprised 7 direct comparisons among 8 interventions. The degree of heterogeneity among these studies was moderate, with* I*^*2*^ value of 54.7% and τ^2^ of 0.2126. The RBF network comprised 8 studies, which included 5 direct comparisons of 6 treatments. This network showed high heterogeneity, with* I*^*2*^ value of 79.5% and τ^2^ of 0.3985. The MR network consisted of 5 studies and comprised 3 direct comparisons among 4 interventions. This network demonstrated high heterogeneity, with* I*^*2*^ value of 89.8% and τ^2^ of 0.4571. The BOP network comprised 8 studies and included 6 direct comparisons among 7 treatments, with low heterogeneity (*I*^*2*^: 6.5%, τ^2^: 7.4592). Finally, the CAL network consisted of 6 studies and included 5 direct comparisons among 6 interventions, with low heterogeneity (*I*^*2*^: 0%, τ^2^: 0).

### Network meta-analysis

**Fig. 4:**
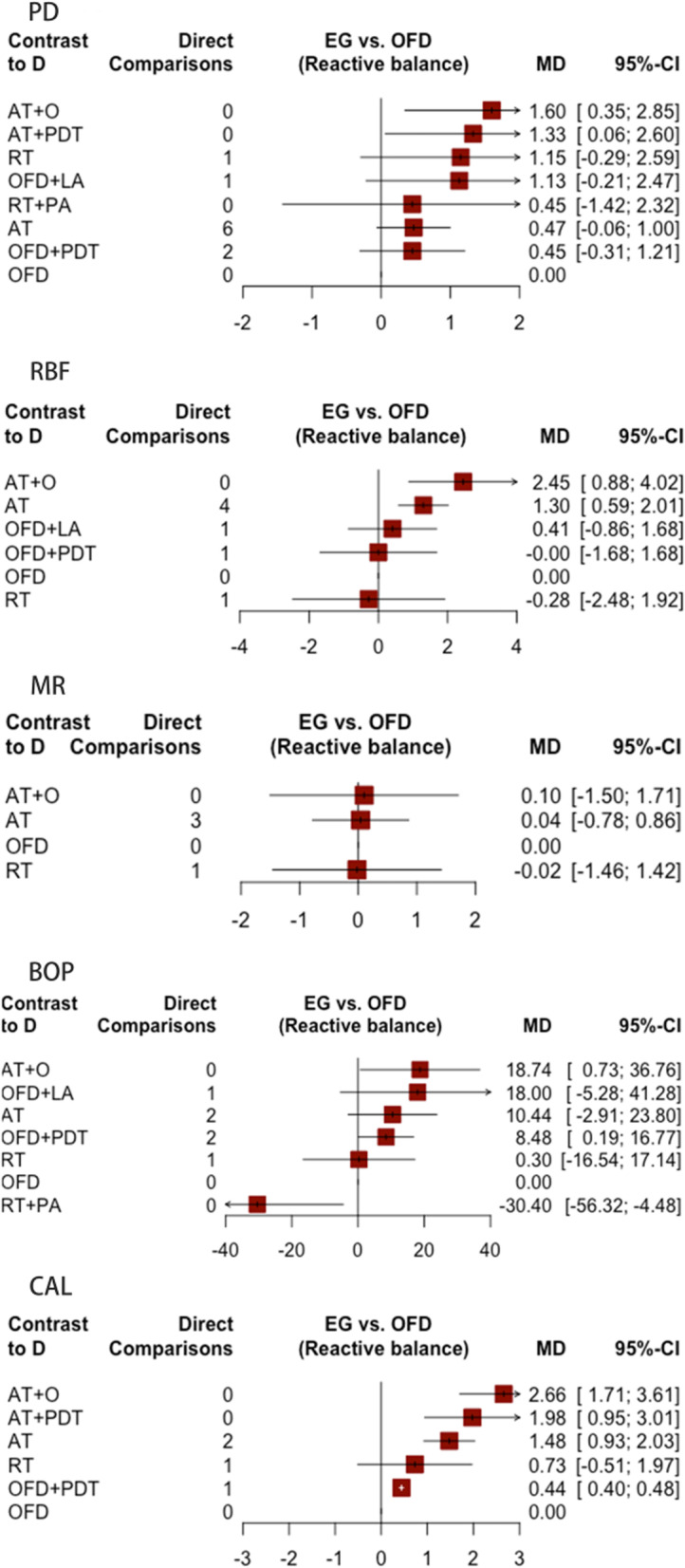
The forest plot comparing the changes in five parameters between OFD and all other treatments

The forest plot (Fig. 4) shows the results of direct and indirect comparisons between OFD and all other treatment methods. In the league tables (Tables 2, 3, 4, 5, and 6), results of the NMA are shown in the lower left of the tables while those of direct comparisons are in the upper right. According to the forest plot and the league tables, we can derive the following findings: 1) augmentative therapy combined with ozone therapy (AT + O) or photodynamic therapy (AT + PDT) reduced PD significantly more than open flap debridement (OFD); 2) increase in bone mass measured by radiographic bone fill (RBF) was significantly higher in AT + O than RT, OFD, OFD + PDT and OFD combined with local antibiotics (OFD + LA), while AT alone was significantly higher than OFD; 3) no significant difference was identified for mucosal recession among the treatment groups; 4) resective therapy combined with phosphoric acid (RT + PA) showed the worst effect on BOP reduction compared to all other treatment groups while AT + O and OFD + PDT showed more reduction than OFD; 5) AT achieved more CAL gain than OFD + PDT and OFD, and OFD + PDT achieved more CAL gain than OFD, all of which were statistically significant. Overall, AT + O showed the most significant response for all parameters.

**Table 2:** League table comparing the changes in PD reduction (mm) between different surgical treatments. The surgical methods were ranked according to the SUCRA value. Numbers outside of brackets indicate mean difference (MD), and numbers inside of brackets are 95% confidence intervals. If MDs is more than 0, column treatment is better. Bold indicates significant results

**AT + O**	**/**	/	/	/	1.13 (-0.01, 2.27)	/	/
0.27 (-1.35, 1.89)	**AT + PDT**	/	/	/	0.86 (-0.29, 2.01)	/	/
0.45 (-1.46, 2.36)	0.18 (-1.74, 2.10)	**RT**	/	0.70 (-0.49, 1.89)	/	/	1.15 (-0.29, 2.59)
0.47 (-1.37, 2.31)	0.20 (-1.65, 2.05)	0.02 (-1.95, 1.99)	**OFD + LA**	/	/	/	1.13 (-0.21, 2.47)
1.15 (-1.11, 3.40)	0.88 (-1.38, 3.14)	0.70 (-0.49, 1.89)	0.68 (-1.63, 2.99)	**RT + PA**	/	/	/
1.13 (-0.01, 2.27)	0.86 (-0.29, 2.01)	0.68 (-0.86, 2.22)	0.66 (-0.78, 2.10)	-0.02 (-1.97, 1.93)	**AT**	/	0.47 (-0.06, 1.00)
1.15 (-0.32, 2.61)	0.88 (-0.60, 2.36)	0.70 (-0.93, 2.33)	0.68 (-0.86, 2.22)	-0.00 (-2.02, 2.02)	0.02 (-0.90, 0.94)	**OFD + PDT**	0.45 (-0.31, 1.21)
**1.60 (0.35, 2.85)**	**1.33 (0.06, 2.60)**	1.15 (-0.29, 2.59)	1.13 (-0.21, 2.47)	0.45 (-1.42, 2.32)	0.47 (-0.06, 1.00)	0.45 (-0.31, 1.21)	**OFD**

**Table 3:** League table comparing the changes in RBF gain (mm) between different surgical treatments. The surgical methods were ranked according to the SUCRA value. Numbers outside of brackets indicate mean difference (MD), and numbers inside of brackets are 95% confidence intervals. If MDs is more than 0, column treatment is better. Bold indicates significant results

**AT + O**	1.15 (-0.25, 2.55)	/	/	/	/
1.15 (-0.25; 2.55)	**AT**	/	/	1.30 (0.59, 2.01)	/
**2.04 (0.02, 4.06)**	0.89 (-0.57, 2.35)	**OFD + LA**	/	0.41 (-0.86, 1.68)	/
**2.45 (0.15, 4.75)**	1.30 (-0.53, 3.13)	0.41 (-1.70, 2.52)	**OFD + PDT**	0.00 (-1.68, 1.68)	/
**2.45 (0.88, 4.02)**	**1.30 (0.59, 2.01)**	0.41 (-0.86, 1.68)	0.00 (-1.68, 1.68)	**OFD**	0.28 (-1.92, 2.48)
**2.73 (0.03, 5.43)**	1.58 (-0.73, 3.89)	0.69 (-1.85, 3.23)	0.28 (-2.49, 3.05)	0.28 (-1.92, 2.48)	**RT**

**Table 4:** League table comparing the changes in MR (mm) between different surgical treatments. The surgical methods were ranked according to the SUCRA value. Numbers outside of brackets indicate mean difference (MD), and numbers inside of brackets are 95% confidence intervals. If MDs is more than 0, column treatment is better. Bold indicates significant results

**AT + O**	0.06 (-1.32, 1.44)	/	/
0.06 (-1.32, 1.44)	**AT**	0.04 (-0.78, 0.86)	/
0.10 (-1.50, 1.71)	0.04 (-0.78, 0.86)	**OFD**	0.02 (-1.42, 1.46)
0.12 (-2.03, 2.27)	0.06 (-1.59, 1.71)	0.02 (-1.42, 1.46)	**RT**

**Table 5:** League table comparing the changes in BOP reduction (%) between different surgical treatments. The surgical methods were ranked according to the SUCRA value. Numbers outside of brackets indicate mean difference (MD), and numbers inside of brackets are 95% confidence intervals. If MDs is more than 0, column treatment is better. Bold indicates significant results

**AT + O**	/	8.30 ( -3.79, 20.39)	/	/	/	/
0.74 (-28.70, 30.18)	**OFD + LA**	/	/	/	18.00 ( -5.28, 41.28)	/
8.30 ( -3.79, 20.39)	7.56 (-19.28, 34.40)	**AT**	/	/	10.44 ( -2.91, 23.80)	**30.70 (11.00, 50.40)**
10.27 ( -9.57, 30.10)	9.52 (-15.19, 34.24)	1.97 (-13.75, 17.68)	**OFD + PDT**	/	**8.48 (0.19, 16.77)**	/
18.44 ( -6.22, 43.11)	17.70 (-11.04, 46.44)	10.14 (-11.35, 31.64)	8.18 (-10.60, 26.95)	**RT**	0.30 (-16.54, 17.14)	/
**18.74 (0.73, 36.76)**	18.00 ( -5.28, 41.28)	10.44 ( -2.91, 23.80)	**8.48 (0.19, 16.77)**	0.30 (-16.54, 17.14)	**OFD**	/
**39.00 (15.88, 62.12)**	**38.26 (4.96, 71.55)**	**30.70 (11.00, 50.40)**	**28.73 (3.53, 53.94)**	20.56 ( -8.60, 49.72)	20.26 ( -3.54, 44.06)	**RT + PA**

**Table 6:** League table comparing changes in CAL gain (mm) between different surgical treatments. The surgical methods were ranked according to the SUCRA value. Numbers outside of brackets indicate mean difference (MD), and numbers inside of brackets are 95% confidence intervals. If MDs is more than 0, column treatment is better. Bold indicates significant results

**AT + O**	**/**	1.18 (0.40, 1.96)	/	/	/
0.68 (-0.49, 1.85)	**AT + PDT**	0.50 (-0.37, 1.37)	/	/	/
**1.18 (0.40, 1.96)**	0.50 (-0.37, 1.37)	**AT**	/	/	**1.48 (0.93, 2.03)**
**1.93 (0.37, 3.49)**	1.25 (-0.36, 2.86)	0.75 (-0.61, 2.10)	**RT**	/	0.73 (-0.51, 1.97)
**2.22 (1.26, 3.17)**	**1.54 (0.51, 2.57)**	**1.04 (0.49, 1.59)**	0.29 (-0.95, 1.53)	**OFD + PDT**	**0.44 (0.40, 0.48)**
**2.66 (1.71, 3.61)**	**1.98 (0.95, 3.01)**	**1.48 (0.93, 2.03)**	0.73 (-0.51, 1.97)	**0.44 (0.40, 0.48)**	**OFD**

### SUCRA ranking of all treatment methods

**Fig. 5:**
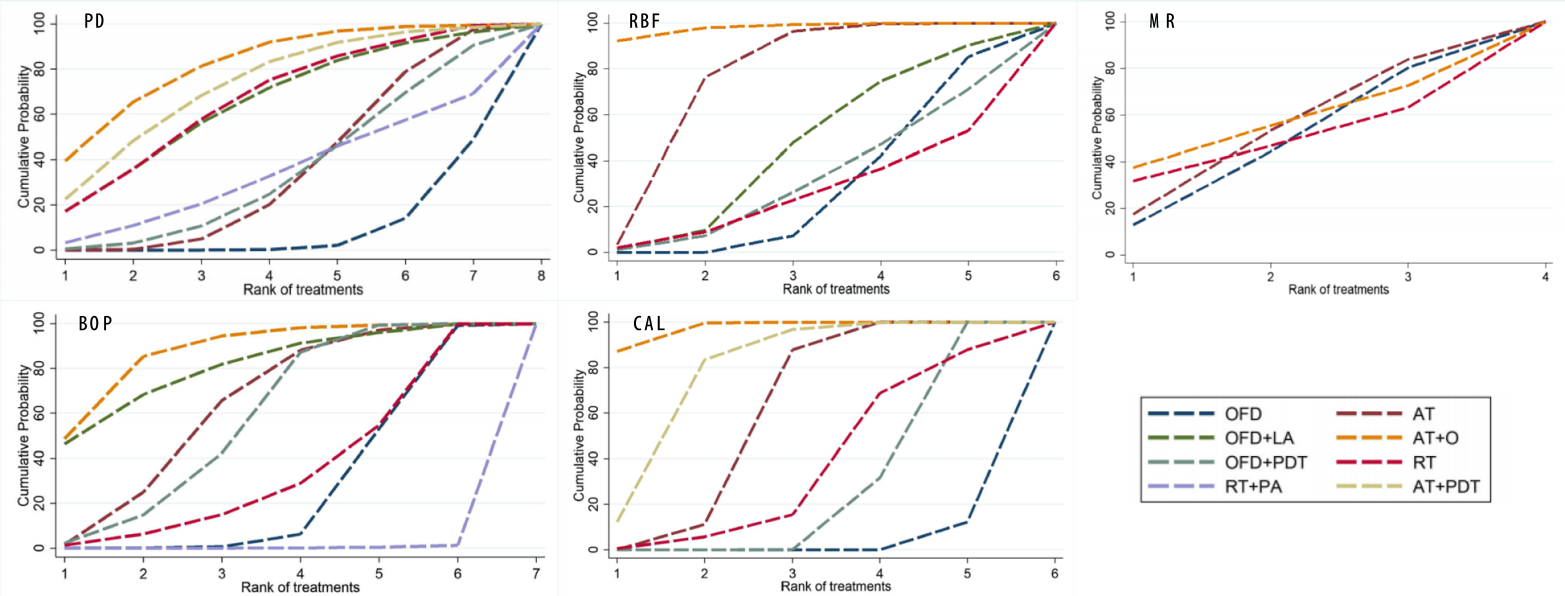
Cumulative ranking curves showing the probability of each intervention method achieving the best outcome for the five parameters evaluated. The SUCRA line displays the relative effectiveness of each treatment considering all possible rankings of treatment effects

The cumulative ranking curves (Fig. 5) and SUCRA ranking table (Appendix 11 Table S9) showed the probabilities of each type of intervention to achieve the evaluated outcomes. AT + O showed the highest probability to achieve the best outcome for all evaluated parameters: most PD reduction (probability 81.6%), most RBF gained (97.8%), most BOP reduction (87.6%) and most CAL gained (97.3%). All surgical procedures were less effective for mucosal retraction, with RT showing slightly higher probability (54.5%) of achieving the best outcome.

## Discussion

### Summary of the main finding

A total of 13 articles with 542 participants were included for this review. Network meta-analysis based on the frequentist method suggests that AT is better than OFD in improving RBF and CAL outcomes, but comparable to OFD in reducing peri-implant soft tissue inflammation. Studies that evaluated MR changes in AT, OFD and RT did not achieve statistical significance. Addition of photodynamic therapy did not significantly alter the effect of AT alone on PD reduction and CAL gain. Similarly, supplementing RT with phosphoric acid did not significantly alter BOP reduction from RT alone. However, the quality of evidence for the above treatments is poor, and the result should thus be interpreted cautiously.

### Effect of augmentative treatment

#### Augmentative treatment alone

Augmentation therapy for peri-implantitis aims to remove granulation tissue and bacterial biofilm to achieve re-osseointegration on the surface of the implant that is previously contaminated. The results of this NMA suggest that AT outperforms OFD in terms of RBF and CAL gain, consistent with previous research [52, 53]. Recent meta-analysis identified a significantly larger marginal bone level gain of 1.7 mm and defect fill (weighted mean difference of 57%) with AT compared with open flap debridement, whereas no differences were identified for clinical measures (PD and BOP reduction) [53]. Thus, regenerative therapy can significantly improve radiographic bone filling but does not outperform OFD in reducing peri-implant soft tissue inflammation. Augmentation therapy also aims to maintain the soft tissue height. But the results of this NMA indicated that there was no statistical difference between AT, OFD, and RT in MR outcomes. This is consistent with animal studies that suggest that peri-implant augmentation surgery does not up-regulate the levels of mid-facial mucosa compared with access flap surgery alone [54, 55]. Thus, whether augmentation surgery can increase the esthetic effect of peri-implantitis remains unclear.

#### Ozone therapy in augmentative treatment

The results of this NMA suggest that AT + O outperforms AT alone in terms of RBF and CAL gain, and PD and BOP reduction. In aqueous and gaseous phases, ozone serves as a reliable and potent antibacterial agent, capable of killing bacteria, fungi, protozoa, and viruses. Current dental practices employ ozone to prevent the development of caries, non-surgical treatment of periodontitis and cleaning of oral restorations, etc [56]. Intraoral gram-positive and gram-negative microorganisms and oral *Streptococcus albicans* can be killed by ozone water [57]. Topical application of ozonated water may facilitate wound healing [58]. Moreover, ozone water has a high level of biocompatibility with human oral epithelium, gingival fibroblasts, as well as periodontal cells [59]. McKenna et al. compared ozone with and without hydrogen peroxide in the treatment of peri-implant mucositis, and found that ozone is capable of significantly reducing plaque, altering gingival and bleeding indexes, with a high potential for peri-implant mucosal management [60]. Furthermore, the antimicrobial effect of ozone therapy potentially delays disease progression, preventing soft tissue friability and thus offering better soft tissue handling if invasive surgery is required. As a result, augmentation therapy with ozone is capable of significantly mitigating peri-implant soft tissue inflammation. Existing research suggests that ozone therapy is capable of facilitating bone formation in autogenous bone grafting and promoting osteogenesis in animal studies [61, 62]. However, the Isler et al. study included in our analysis is the only study on the use of ozone therapy in surgical management of peri-implantitis [49], and our attention should be paid to this finding.

#### Photodynamic therapy in augmentative therapy

Photodynamic therapy uses low energy visible light to stimulate a non-toxic light-sensitive dye called a “photosensitizer” (PS) [63]. This results in formation of singlet oxygen free radicals, which are toxic to bacteria and cells [64]. It has been used repeatedly for treating periodontitis and peri-implantitis. In this study, PDT combined with AT did not show significant difference in PD reduction and CAL gain compared with AT alone. Vohra et al. conducted a meta-analysis which suggested that PDT improves bone implant contact and re-osseointegration but does not differ from chemical debridement [65]. Further meta-analyses suggested that PDT improves peri-implantitis outcomes [66]. Since only one study evaluated this treatment method, this finding should be treated cautiously.

### Effect of resective therapy

In this study, RT did not achieve a good therapeutic effect. In terms of BOP, AT + O was significantly lower than RT + PA, and no significant difference was identified between RT and RT + PA. It can be seen that the use of chemicals has not achieved better therapeutic effect. The chemical agents recommended for use on exposed implants after mechanical decontamination during implant surgery include hydrogen peroxide, citric acid, sodium chloride, chloramines, tetracycline hydrochloride, and chlorhexidine gluconate [67]. However, a meta-analysis did not find any method to be superior [68], and 0.2% chlorhexidine gluconate has no better therapeutic effect than sterile saline [69]. Accordingly, the use of chemical agents in surgical resection for peri-implantitis does not improve the final outcome.

### Limitation

As far as we know, this has been the first NMA comparing different surgical methods for peri-implantitis. Nevertheless, there are some limitations to this study. Due to the small number of relevant articles (and their sample sizes), some of the results were subject to bias. Among the included randomized controlled trials, 30.8% were at high risk of bias and only 53.8% were at low risk. The quality of evidence was significantly low, leading to low confidence in the estimation of effect. Thus, while we can show which surgical option is more effective, the optimal surgical option for peri-implantitis remains unclear. Future research should involve well-designed high-quality RCTs with larger sample sizes to accurately address the above limitations.

## Conclusions

Within the limitations of this systematic review and network meta-analysis, our data showed that augmentation surgery is capable of significantly increasing the amount of bone filling on imaging, whereas the control of peri-implant soft tissue inflammation is comparable to open flap debridement. Augmentative surgery combined with ozone therapy is likely to achieve better outcomes but should be implemented with caution as the quality of evidence is poor. The present data do not contribute to the final determination of the optimal surgical option for peri-implantitis.

## Supplementary Information

**Additional file 1.** 

## Data Availability

All data in this study are available within the manuscript.

## Abbreviations

RCTs: Randomized controlled trials
PD: Probing depth
RBF: Radiographic bone fill
MR: Mucosal recession
BOP: Bleeding on probing
CAL: Clinical attachment level
PI: Plaque index
GI: Gingival index
SUP: Suppuration
VDD: Vertical defect depth
BL: Bone level
BI: Bleeding index
KM: Keratinized mucosa
SOP: Suppuration on probing
OFD: Open flap debridement
RT: Resective therapy
AT: Augmentative therapy
OFD + LA: Open flap debridement combined with local antibiotics
OFD + PDT: Open flap debridement combined with photodynamic therapy
RT + PA: Resective therapy combined with phosphoric acid
AT + PDT: Augmentative therapy combined with photodynamic therapy
AT + O: Augmentative therapy combined with ozone therapy
NMA: Network meta-analysis
MD: Mean difference
SUCRA: Surface under Cumulative Ranking Curve
ROB: Risk of bias evaluation
CINeMA: Confidence in Network Meta-Analysis

## References

[1] Esposito M, Hirsch JM, Lekholm U (1998). Biological factors contributing to failures of osseointegrated oral implants.  (I). success criteria and epidemiology. Eur J Oral Sci.

[2] Roos-Jansåker AM, Lindahl C, Renvert H (2006). Nine- to fourteen-year follow-up of implant treatment. part II: presence of peri-implant lesions. J Clin Periodontol.

[3] Liaw K, Delfini RH, Abrahams JJ (2015). Dental implant complications. Semin Ultrasound CT MR.

[4] Derks J, Schaller D, Håkansson J (2016). Peri-implantitis - onset and pattern of progression. J Clin Periodontol.

[5] Schwarz F, Derks J, Monje A (2018). Peri-implantitis. J Clin Periodontol.

[6] Berglundh T, Armitage G, Araujo MG (2018). Peri-implant diseases and conditions: consensus report of workgroup 4 of the 2017 world workshop on the classification of periodontal and peri-implant diseases and conditions. J Clin Periodontol.

[7] Zhang H, Li W, Zhang L (2018). A nomogram prediction of peri-implantitis in treated severe periodontitis patients: a 1-5-year prospective cohort study. Clin Implant Dent Relat Res.

[8] Derks J, Tomasi C. Peri-implant health and disease. a systematic review of current epidemiology. J Clin Periodontol. 2015;42 Suppl 16: S158-S171. 10.1111/jcpe.12334 25495683

[9] Aguirre-Zorzano LA, Estefanía-Fresco R, Telletxea O (2015). Prevalence of peri-implant inflammatory disease in patients with a history of periodontal disease who receive supportive periodontal therapy. Clin Oral Implants Res.

[10] Berglundh T, Jepsen S, Stadlinger B (2019). Peri-implantitis and its prevention. Clin Oral Implants Res.

[11] Esposito M, Grusovin MG, Worthington HV (2012). Interventions for replacing missing teeth: treatment of peri-implantitis. Cochrane Database Syst Rev.

[12] Roccuzzo A, De Ry SP, Sculean A (2020). Current approaches for the non-surgical management of peri-implant diseases. Curr Oral Health Rep.

[13] Suárez-López Del Amo F, Yu SH, Wang HL (2016). Non-surgical therapy for peri-implant diseases: a systematic review. J Oral Maxillofac Res.

[14] Faggion CM, Listl S, Frühauf N (2014). A systematic review and Bayesian network meta-analysis of randomized clinical trials on non-surgical treatments for peri-implantitis. J Clin Periodontol.

[15] Berglundh T, Wennström JL, Lindhe J (2018). Long-term outcome of surgical treatment of peri-implantitis. a 2–11-year retrospective study. Clin Oral Implants Res.

[16] Schwarz F, Jepsen S, Obreja K (2022). Surgical therapy of peri-implantitis. Periodontol 2000.

[17] Hallström H, Persson GR, Lindgren S (2017). Open flap debridement of peri-implantitis with or without adjunctive systemic antibiotics: a randomized clinical trial. J Clin Periodontol.

[18] Papadopoulos CA, Vouros I, Menexes G (2015). The utilization of a diode laser in the surgical treatment of peri-implantitis. a randomized clinical trial. Clin Oral Investig.

[19] Keeve PL, Koo KT, Ramanauskaite A (2019). Surgical treatment of periimplantitis with non-augmentative techniques. Implant Dent.

[20] Aljohani M, Yong SL, Bin RA (2020). The effect of surgical regenerative treatment for peri-implantitis: a systematic review. Saudi Dent J.

[21] Jepsen S, Schwarz F, Cordaro L, et al. Regeneration of alveolar ridge defects. consensus report of group 4 of the 15th European workshop on periodontology on bone regeneration. J Clin Periodontol. 2019;46 Suppl 21:277–286. 10.1111/jcpe.13121 31038223

[22] Ramanauskaite A, Fretwurst T, Schwarz F (2021). Efficacy of alternative or adjunctive measures to conventional non-surgical and surgical treatment of peri-implant mucositis and peri-implantitis: a systematic review and meta-analysis. Int J Implant Dent.

[23] Chan HL, Lin GH, Suarez F (2014). Surgical management of peri-implantitis: a systematic review and meta-analysis of treatment outcomes. J Periodontol.

[24] Sahrmann P, Attin T, Schmidlin PR (2011). Regenerative treatment of peri-implantitis using bone substitutes and membrane: a systematic review. Clin Implant Dent Relat Res.

[25] Khoury F, Keeve PL, Ramanauskaite A, et al. Surgical treatment of peri-implantitis - consensus report of working group 4. Int Dent J. 2019;69 Suppl 2(Suppl 2):18–22. 10.1111/idj.12505 PMC9379045 31478576

[26] Faggion CM, Chambrone L, Listl S (2013). Network meta-analysis for evaluating interventions in implant dentistry: the case of peri-implantitis treatment. Clin Implant Dent Relat Res.

[27] Higgins JP, Altman DG, Gøtzsche PC, et al. The Cochrane Collaboration’s tool for assessing risk of bias in randomised trials. BMJ. 2011;343: d5928. 10.1136/bmj.d5928 PMC3196245 22008217

[28] Hutton B, Salanti G, Caldwell DM (2015). The PRISMA extension statement for reporting of systematic reviews incorporating network meta-analyses of health care interventions: checklist and explanations. Ann Intern Med.

[29] Shim S, Yoon BH, Shin IS (2017). Network meta-analysis: application and practice using Stata. Epidemiol Health.

[30] Rücker G (2012). Network meta-analysis, electrical networks and graph theory. Res Synth Methods.

[31] Shim SR, Kim SJ, Lee J (2019). Network meta-analysis: application and practice using R software. Epidemiol Health.

[32] Dias S, Welton NJ, Caldwell DM (2010). Checking consistency in mixed treatment comparison meta-analysis. Stat Med.

[33] Higgins JP, Thompson SG, Deeks JJ (2003). Measuring inconsistency in meta-analyses. BMJ.

[34] Hu ML, Zheng G, Lin H (2021). Network meta-analysis of the treatment efficacy of different lasers for peri-implantitis. Lasers Med Sci.

[35] Mbuagbaw L, Rochwerg B, Jaeschke R, et al. Approaches to interpreting and choosing the best treatments in network meta-analyses. Syst Rev. 2017;6(1):79. Published 2017 Apr 12. 10.1186/s13643-017-0473-z PMC5389085 28403893

[36] Sterne JAC, Savović J, Page MJ (2019). RoB 2: a revised tool for assessing risk of bias in randomised trials. BMJ.

[37] Salanti G, Del Giovane C, Chaimani A (2014). Evaluating the quality of evidence from a network meta-analysis. PLoS ONE.

[38] Nikolakopoulou A, Higgins JPT, Papakonstantinou T (2020). CINeMA: an approach for assessing confidence in the results of a network meta-analysis. PLoS Med.

[39] Renvert S, Giovannoli JL, Roos-Jansåker AM (2021). Surgical treatment of peri-implantitis with or without a deproteinized bovine bone mineral and a native bilayer collagen membrane: a randomized clinical trial. J Clin Periodontol.

[40] Renvert S, Roos-Jansåker AM, Persson GR (2018). Surgical treatment of peri-implantitis lesions with or without the use of a bone substitute-a randomized clinical trial. J Clin Periodontol.

[41] Jepsen K, Jepsen S, Laine ML (2016). Reconstruction of peri-implant osseous defects: a multicenter randomized trial. J Dent Res.

[42] Hamzacebi B, Oduncuoglu B, Alaaddinoglu EE (2015). Treatment of peri-implant bone defects with platelet-rich fibrin. Int J Periodontics Restorative Dent.

[43] Emanuel N, Machtei EE, Reichart M (2020). D-PLEX500: a local biodegradable prolonged release doxycycline-formulated bone graft for the treatment for peri-implantitis.  a randomized controlled clinical study. Quintessence Int.

[44] Cha JK, Lee JS, Kim CS (2019). Surgical therapy of peri-implantitis with local minocycline: a 6-month randomized controlled clinical trial. J Dent Res.

[45] Albaker AM, ArRejaie AS, Alrabiah M (2018). Effect of antimicrobial photodynamic therapy in open flap debridement in the treatment of peri-implantitis: a randomized controlled trial. Photodiagnosis Photodyn Ther.

[46] Bombeccari GP, Guzzi G, Gualini F (2013). Photodynamic therapy to treat periimplantitis. Implant Dent.

[47] Hentenaar DFM, De Waal YCM, Strooker H (2017). Implant decontamination with phosphoric acid during surgical peri-implantitis treatment: a RCT. Int J Implant Dent.

[48] Rakašević D, Lazić Z, Rakonjac B (2016). Efficiency of photodynamic therapy in the treatment of peri-implantitis – a three-month randomized controlled clinical trial. Srp Arh Celok Lek.

[49] Isler SC, Unsal B, Soysal F (2018). The effects of ozone therapy as an adjunct to the surgical treatment of peri-implantitis. J Periodontal Implant Sci.

[50] Wohlfahrt JC, Lyngstadaas SP, Rønold HJ (2012). Porous titanium granules in the surgical treatment of peri-implant osseous defects: a randomized clinical trial. Int J Oral Maxillofac Implants.

[51] Lasserre JF, Brecx MC, Toma S (2020). Implantoplasty versus glycine air abrasion for the surgical treatment of peri-implantitis: a randomized clinical trial. Int J Oral Maxillofac Implants.

[52] Madi M, Htet M, Zakaria O (2018). Re-osseointegration of dental implants after periimplantitis treatments: a systematic review. Implant Dent.

[53] Tomasi C, Regidor E, Ortiz-Vigón A, et al. Efficacy of reconstructive surgical therapy at peri-implantitis-related bone defects. a systematic review and meta-analysis. J Clin Periodontol. 2019;46 Suppl 21:340–356. 10.1111/jcpe.13070 30667523

[54] Ramos UD, Suaid FA, Wikesjö UME (2017). Comparison between two antimicrobial protocols with or without guided bone regeneration in the treatment of peri-implantitis. a histomorphometric study in dogs. Clin Oral Implants Res.

[55] Almohandes A, Carcuac O, Abrahamsson I (2019). Re-osseointegration following reconstructive surgical therapy of experimental peri-implantitis. a pre-clinical in vivo study. Clin Oral Implants Res.

[56] Azarpazhooh A, Limeback H (2008). The application of ozone in dentistry: a systematic review of literature. J Dent.

[57] Nagayoshi M, Fukuizumi T, Kitamura C (2004). Efficacy of ozone on survival and permeability of oral microorganisms. Oral Microbiol Immunol.

[58] Romary DJ, Landsberger SA, Bradner KN, et al. Liquid ozone therapies for the treatment of epithelial wounds: a systematic review and meta-analysis. Int Wound J. 2022. 10.1111/iwj.13941 PMC10031250 36056800

[59] Huth KC, Jakob FM, Saugel B (2006). Effect of ozone on oral cells compared with established antimicrobials. Eur J Oral Sci.

[60] McKenna DF, Borzabadi-Farahani A, Lynch E (2013). The effect of subgingival ozone and/or hydrogen peroxide on the development of peri-implant mucositis: a double-blind randomized controlled trial. Int J Oral Maxillofac Implants.

[61] Ozdemir H, Toker H, Balcı H, Ozer H (2013). Effect of ozone therapy on autogenous bone graft healing in calvarial defects: a histologic and histometric study in rats. J Periodontal Res.

[62] Alpan AL, Toker H, Ozer H (2016). Ozone therapy enhances osseous healing in rats with diabetes With calvarial defects: a morphometric and immunohistochemical study. J Periodontol.

[63] Dai T, Fuchs BB, Coleman JJ (2012). Concepts and principles of photodynamic therapy as an alternative antifungal discovery platform. Front Microbiol.

[64] de Oliveira RR, Schwartz-Filho HO, Novaes AB, Taba M (2007). Antimicrobial photodynamic therapy in the non-surgical treatment of aggressive periodontitis: a preliminary randomized controlled clinical study. J Periodontol.

[65] Vohra F, Al-Rifaiy MQ, Lillywhite G, Abu Hassan MI, Javed F (2014). Efficacy of mechanical debridement with adjunct antimicrobial photodynamic therapy for the management of peri-implant diseases: a systematic review. Photochem Photobiol Sci.

[66] Sivaramakrishnan G, Sridharan K (2018). Photodynamic therapy for the treatment of peri-implant diseases: a network meta-analysis of randomized controlled trials. Photodiagnosis Photodyn Ther.

[67] Renvert S, Polyzois I (2018). Treatment of pathologic peri-implant pockets. Periodontol 2000.

[68] Claffey N, Clarke E, Polyzois I, Renvert S (2008). Surgical treatment of peri-implantitis. J Clin Periodontol.

[69] Carcuac O, Derks J, Abrahamsson I (2017). Surgical treatment of peri-implantitis: 3-year results from a randomized controlled clinical trial. J Clin Periodontol.

