# Supplementary material for: Efficacy of surgical methods for peri-implantitis: a systematic review and network meta-analysis
Source: BMC Oral Health. 2023 Apr 19;23:227. doi: 10.1186/s12903-023-02956-6 (PMC10116816; doi:10.1186/s12903-023-02956-6)
Supplement: Supplementary file 1 — Additional file 1. [file 12903_2023_2956_MOESM1_ESM.docx]

**Appendix**

**Appendix 1: PRIMA checklist**

| section and topic | Item# | Checkliste item | Reported on Page # |
| --- | --- | --- | --- |
| **Title** |  |  |  |
| Title | 1 | Efficacy of surgical methods for peri-implantitis: a systematic review and network meta-analysis | 1 |
| **Abstract** |  |  |  |
| Structured summary | 2 | Provide a structured summary including, as applicable: **Background**: main objectives. **Methods**: data sources; study eligibility criteria, participants, and interventions; study appraisal; and synthesis methods, such as network meta-analysis. **Results**: number of studies and participants identified; summary estimates with corresponding confidence/credible intervals. **Discussion/Conclusions**: limitations; conclusions and implications of findings. **Other**: primary source of funding; systematic review registration number with registry name. | 2 |
| **Introduction** |  |  |  |
| Rational | 3 | Describe the rationale for the review in the context of existing knowledge. | 2-4 |
| Objectives | 4 | Provide an explicit statement of the objective(s) or question(s) the review addresses. | 4-5 |
| **Methos** |  |  |  |
| Registration and protocol | 5 | Provide registration information for the review, including register name and registration number, or state that the review was not registered. | 6 |
| Eligibility criteria | 6 | Specify the inclusion and exclusion criteria for the review and how studies were grouped for the syntheses. | 6-8 |
| Information sources | 7 | Specify all databases, registers, websites, organisations, reference lists and other sources searched or consulted to identify studies. Specify the date when each source was last searched or consulted. | 8 |
| Search strategy | 8 | Present the full search strategies for all databases, registers and websites, including any filters and limits used. | Appendix 2 |
| Selection process | 9 | Specify the methods used to decide whether a study met the inclusion criteria of the review, including how many reviewers screened each record and each report retrieved, whether they worked independently, and if applicable, details of automation tools used in the process. | 8 |
| Data collection process | 10 | Specify the methods used to collect data from reports, including how many reviewers collected data from each report, whether they worked independently, any processes for obtaining or confirming data from study investigators, and if applicable, details of automation tools used in the process. | 8-9 |
| Data items | 11 | List and define all outcomes for which data were sought. Specify whether all results that were compatible with each outcome domain in each study were sought (e.g. for all measures, time points, analyses), and if not, the methods used to decide which results to collect. | 7 |
| Geometry of the network | S1 | Describe methods used to explore the geometry of the treatment network under study and potential biases related to it. This should include how the evidence base has been graphically summarized for presentation, and what characteristics were compiled and used to describe the evidence base to readers. | 9-10 |
| Study risk of bias assessment | 12 | Specify the methods used to assess risk of bias in the included studies, including details of the tool(s) used, how many reviewers assessed each study and whether they worked independently, and if applicable, details of automation tools used in the process. | 10 |
| Summary measures | 13 | State the principal summary measures (eg, risk ratio, difference in means). Also describe the use of additional summary measures assessed, such as treatment rankings and surface under the cumulative ranking curve (SUCRA) values, as well as modified approaches used to present summary findings from meta-analyses. | 10 |
| Planned methods of analysis | 14 | Describe the methods of handling data and combining results of studies for each network meta-analysis. This should include, but not be limited to: • Handling of multi-arm trials; · Selection of variance structure; · Selection of prior distributions in Bayesian analyses; and · Assessment of model fit. | 9-10 |
| Assessment of Inconsistency | S2 | Describe the statistical methods used to evaluate the agreement of direct and indirect evidence in the treatment network(s) studied. Describe efforts taken to address its presence when found. | 9-10 |
| Reporting bias assessment | 15 | Describe any methods used to assess risk of bias due to missing results in a synthesis (arising from reporting biases). | 10 |
| Additional analyses | 16 | Describe methods of additional analyses if done, indicating which were pre-specified. This may include, but not be limited to, the following: · Sensitivity or subgroup analyses; · Meta-regression analyses; · Alternative formulations of the treatment network; and · Use of alternative prior distributions for Bayesian analyses (if applicable) | NA |
| **Results** |  |  |  |
| Study selection | 17 | Give numbers of studies screened, assessed for eligibility, and included in the review, with reasons for exclusions at each stage, ideally with a flow diagram. | 11 |
| Presentation of network structure | S3 | Provide a network graph of the included studies to enable visualization of the geometry of the treatment network. | Fig. 3 |
| Summary of network geometr | S4 | Provide a brief overview of characteristics of the treatment network. This may include commentary on the abundance of trials and randomized patients for the different interventions and pairwise comparisons in the network, gaps of evidence in the treatment network, and potential biases reflected by the network structure. | 15 |
| Study characteristics | 18 | For each study, present characteristics for which data were extracted (eg, study size, PICOS, follow-up period) and provide the citations. | 12-13 |
| Risk of bias within studies | 19 | Present data on risk of bias of each study and, if available, any outcome level assessment | 14-15 |
| Results of individual studies | 20 | For all outcomes considered (benefits or harms), present, for each study: (1) simple summary data for each intervention group, and (2) effect estimates and confidence intervals. Modified approaches may be needed to deal with information from larger networks. | 16 |
| Synthesis of results | 21 | Present results of each meta-analysis done, including confidence/credible intervals. In larger networks, authors may focus on comparisons versus a particular comparator (eg, placebo or standard care), with full findings presented in an appendix. League tables and forest plots may be considered to summarize pairwise comparisons. If additional summary measures were explored (such as treatment rankings), these should also be presented. | 16 |
| Exploration for inconsistency | S5 | Describe results from investigations of inconsistency. This may include such information as measures of model fit to compare consistency and inconsistency models, P values from statistical tests, or summary of inconsistency estimates from different parts of the treatment network. | 15 |
| Risk of bias in studies | 22 | Present assessments of risk of bias for each included study. | 14 |
| Results of additional analyses | 23 | Give results of additional analyses, if done (eg, sensitivity or subgroup analyses, metaregression analyses, alternative network geometries studied, alternative choice of prior distributions for Bayesian analyses, and so forth). | NA |
| **Discussion** |  |  |  |
| Summary of evidence | 24 | Summarize the main findings, including the strength of evidence for each main outcome; consider their relevance to key groups (eg, health care providers, users, and policy makers) | 21-24 |
| Limitations | 25 | Discuss limitations at study and outcome level (eg, risk of bias), and at review level (eg, incomplete retrieval of identified research, reporting bias). Comment on the validity of the assumptions, such as transitivity and consistency. Comment on any concerns regarding network geometry (eg, avoidance of certain comparisons). | 24-25 |
| Conclusions | 26 | Provide a general interpretation of the results in the context of other evidence, and implications for future research. | 25 |
| **Other information** |  |  |  |
| Support | 27 | Describe sources of financial or non-financial support for the review, and the role of the funders or sponsors in the review. | 25-28 |

**Appendix 2: search strategy**

2.1 Pubmed

#1 Search: (peri-implantitis [MeSH Terms]) AND (surgery)

"peri implantitis"[MeSH Terms] AND ("surgery"[MeSH Subheading] OR "surgery"[All Fields] OR "surgical procedures, operative"[MeSH Terms] OR ("surgical"[All Fields] AND "procedures"[All Fields] AND "operative"[All Fields]) OR "operative surgical procedures"[All Fields] OR "general surgery"[MeSH Terms] OR ("general"[All Fields] AND "surgery"[All Fields]) OR "general surgery"[All Fields] OR "surgery s"[All Fields] OR "surgerys"[All Fields] OR "surgeries"[All Fields]) (897)

2.2 EMBASE

No. Query Results Results

#3. #1 AND #2 1,924

#2. 'surgery'/exp OR 'surgery' 7,593,957

#1. 'periimplantitis'/exp OR 'periimplantitis' 3,729

2.3 Web of science

#1 (TS=(peri-implantitis)) AND TS=(surgery) (1077)

2.4 Cochrane

#1 - ("peri-implantitis"): ti,ab,kw AND (surgery):ti,ab,kw (632)

2.5 Hand search

The following journals were hand-searched: Journal of Dental Research, Journal of Clinical Periodontology, Journal of Periodontology, Clinical Oral Implants Research, Clinical Implant Dentistry and Related Research, The International Journal of Oral & Maxillofacial Implants, Journal of Oral and Maxillofacial Surgery, International Journal of Oral Implantology, Clinical Oral Investigations, and International Journal of Periodontics and Restorative Dentistry。

**Appendix 3: PRIMA Flow Diagram**


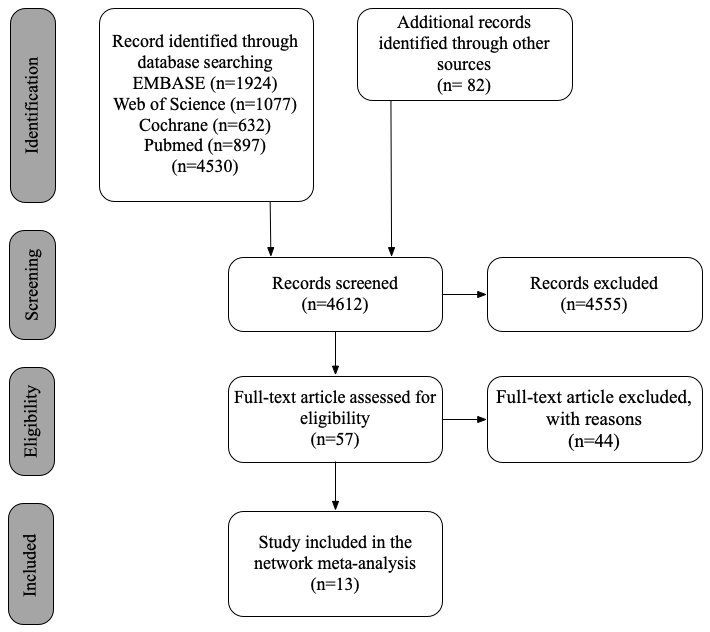


**Figure S1.** The search process

**Appendix 4: List of included studies**

| Publication | Country | Follow-up times (months) | Participant age (years) | Control group | Test group | Number of intervention and comparison | Outcomes |
| --- | --- | --- | --- | --- | --- | --- | --- |
| Renvert S (2021)^40^ | Sweden, France, Germany | 12 | CG: 62.9±13.0 TG: 62.2±10.2 | OFD | AT | 32/34 | BL/PD/BI/SUP/REC |
| Cha JK (2019)^46^ | South Korea | 6 | 61.6±21.6 | OFD | OFD + LA | 25/25 | PD/PI/GI |
| Renvert S (2018)^41^ | Sweden | 12 | CG: 70 ±7.8 TG: 67.5±11.3 | OFD | AT | 20/21 | PD/BL |
| Isler SC (2018)^51^ | Turkey | 12 | CG: 54.18±10.36 TG: 54.4±8.08 | AT | AT + O | 21/20 | PI/GI/BOP/PD/CAL/REC/VDD |
| Albaker AM (2018)^47^ | Saudi Arabia | 12 | CG: 61.5±9.9 TG: 58.4±8.0 | OFD | OFD + PDT | 13/11 | PI/BOP/PD/BL |
| Hentenaar DFM (2017)^49^ | Netherlands | 3 | CG: 57.0±13.7 TG: 60.9±7.2 | RT | RT + PA | 20/30 | BOP/SUP/PD |
| Rakašević D (2016)^50^ | Serbia | 3 | CG: 60 TG: 57.59 | AT | AT + PDT | 19/21 | PD/CAL/BOP |
| Jepsen K (2016)^42^ | Germany | 12 | 58.4±12.3 | OFD | AT | 26/33 | VDD/PD/BOP/PI |
| Hamzacebi B (2015)^43^ | Turkey | 6 | 60.98±11.9 | OFD | AT | 19/19 | BOP/PD/REC/CAL/KM |
| Bombeccari GP (2013)^48^ | Italy | 6 | 46±13.0 | OFD | OFD + PDT | 20/20 | PD/CAL/BOP |
| Wohlfahrt JC (2012)^44^ | Norway | 12 | CG: 65.0±10.0 TG: 57.2±12.3 | OFD | RT | 16/16 | PD/BOP |
| Emanuel N (2012)^45^ | Israel | 12 | 64.81±7.61 | OFD | AT | 14/18 | PD/CAL/BL/BOP/REC |
| Lasserre JF (2020)^52^ | Belgium | 6 | 66.5±24.5 | RT | OFD | 15/14 | PI/BOP/SOP/PD/CAL/REC/BL |

**Table S1.** Characteristics of the included articles and their interventions. OFD: open flap debridement; AT: augmentative therapy; RT: resective therapy; PD: probing depth; CAL：Clinical attachment level; PI: Plaque Index; BOP: Bleeding on probing; GI: Gingival index; REC: mucosal recession; SUP: suppuration; VDD: vertical defect depth;

**Appendix 5: Full-text articles excluded, with reasons**

| Main reason for exclusion | Study |
| --- | --- |
| Not an RCT (n=17) | Lerario (2016), Mercado (2018), Bianchini (2018), Englezos (2018), Dalago (2019), Bianchini (2020), Ravida (2020), Ramanoskaite (2018), Nart (2017), Matarasso (2014), Schwarz (2014), Galarraga-Vinueza（2020）, Roos-Jansåker(2011),Roos-Jansåker(2007), Benhneck (2000),Khoury (2001), Roccuzzo (2011) |
| replaced by the sanme study which has the longest follow-up. (n=4) | Isehed (2016), Schwarz (2013), Schwarz (2012), Schwarz (2011) |
| Unable to extract data (n=1) | Papadopoulos (2015) |
| Mixture of therapies (non-surgical and surgical) (n=3) | Carcuac (2017) ，De Angelis (2012) ，Esposito(2013) |
| Subgroups not appropriate for this study (n=19) | Hallström (2017), Teughels (2021), Solonko (2022), Polymeri (2020), de Tapia(2019), Arab (2016), de Waal (2015), [Aghazadeh](https://pubmed.ncbi.nlm.nih.gov/?size=50&term=Aghazadeh+A&cauthor_id=22548359) (2012), Schwarz (2009), Romeo (2005), Romeo(2008), Stewart (2018), de Waal (2013), Schwarz (2010), Isehed (2016), Schwarz (2014), Wohlfahrt (2012), Wang (2021), Esposito(2013) |

**Table S2.** Network meta-analysis eligible comparisons.

**Appendix 6:** Evaluation of heterogeneity and inconsistency

| Outcomes | *I^2^*(%) | τ^2^ | Q-value |
| --- | --- | --- | --- |
| PD | 54.7 | 0.2126 | 13.24 |
| RBF | 79.5 | 0.3985 | 14.61 |
| MR | 89.8 | 0.4571 | 19.55 |
| BOP | 6.5 | 7.4592 | 2.14 |
| CAL | 0 | 0 | 0 |

**Table S3.** Quantifying heterogeneity and inconsistency

**Appendix 7: network plots**


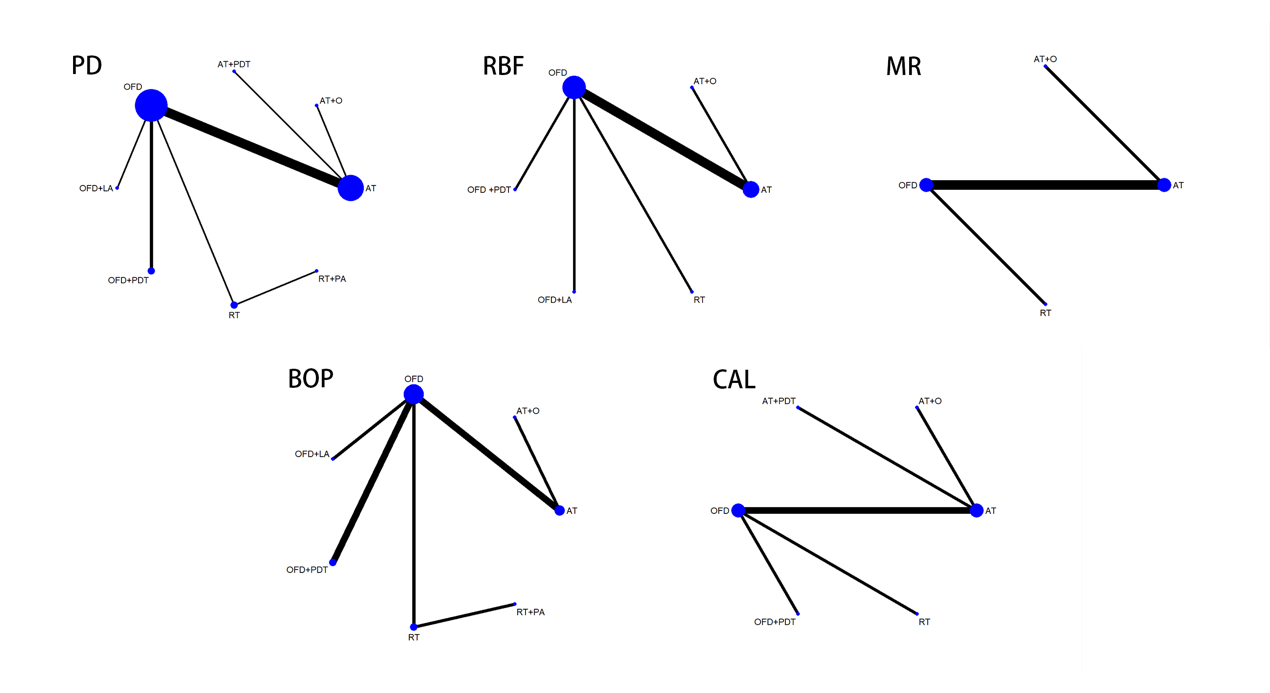


**Figure S2.** Interpretation of Network Plots: The size of the nodes represents the number of patients who will participate in each treatment. A line indicates a direct comparison between the two approaches, and the thickness of the line indicates the number of studies.

**Appendix 8:** **comparison-adjusted funnel plots**


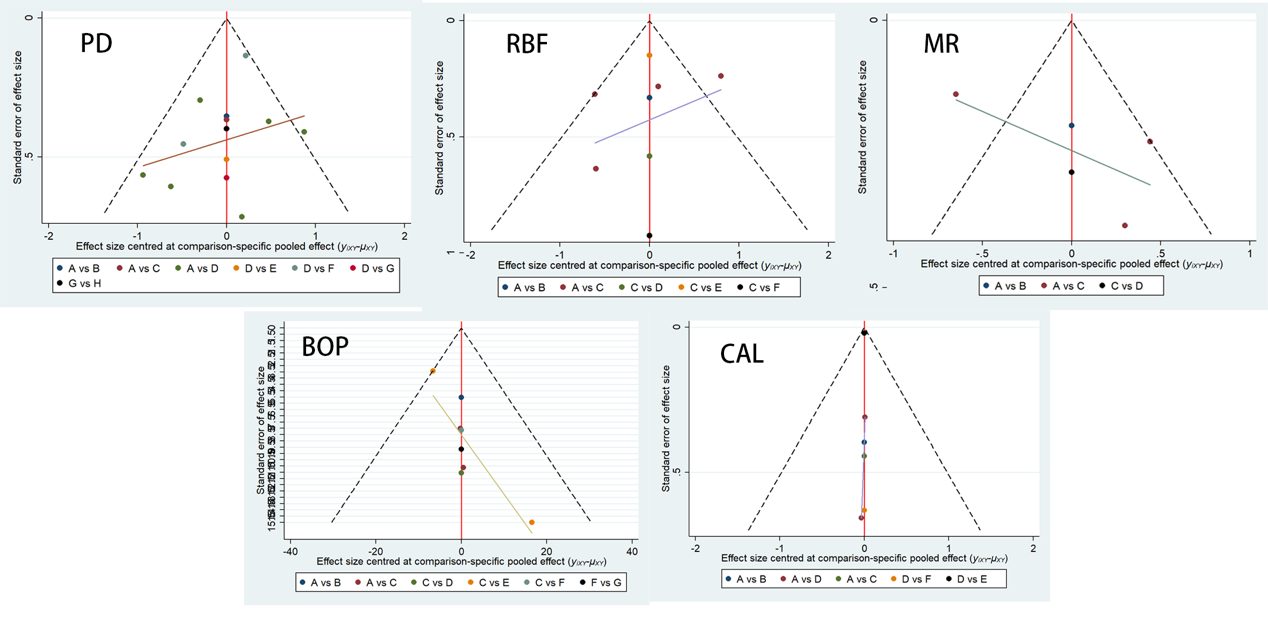


**Figure S3.** Comparison-adjusted funnel spots are used to evaluate the clinical efficacy of different surgical methods in the treatment of peri-implantitis in terms of probing depth (PD), radiographic bone fill (RBF), mucosal recession (MR), bleeding of probing (BOP), clinical attach level (CAL)

**Appendix 9: Forest plots**

Forest Plot interpretation: All treatments were compared with OFD, and treatments across the Y-axis were not statistically significant with OFD.


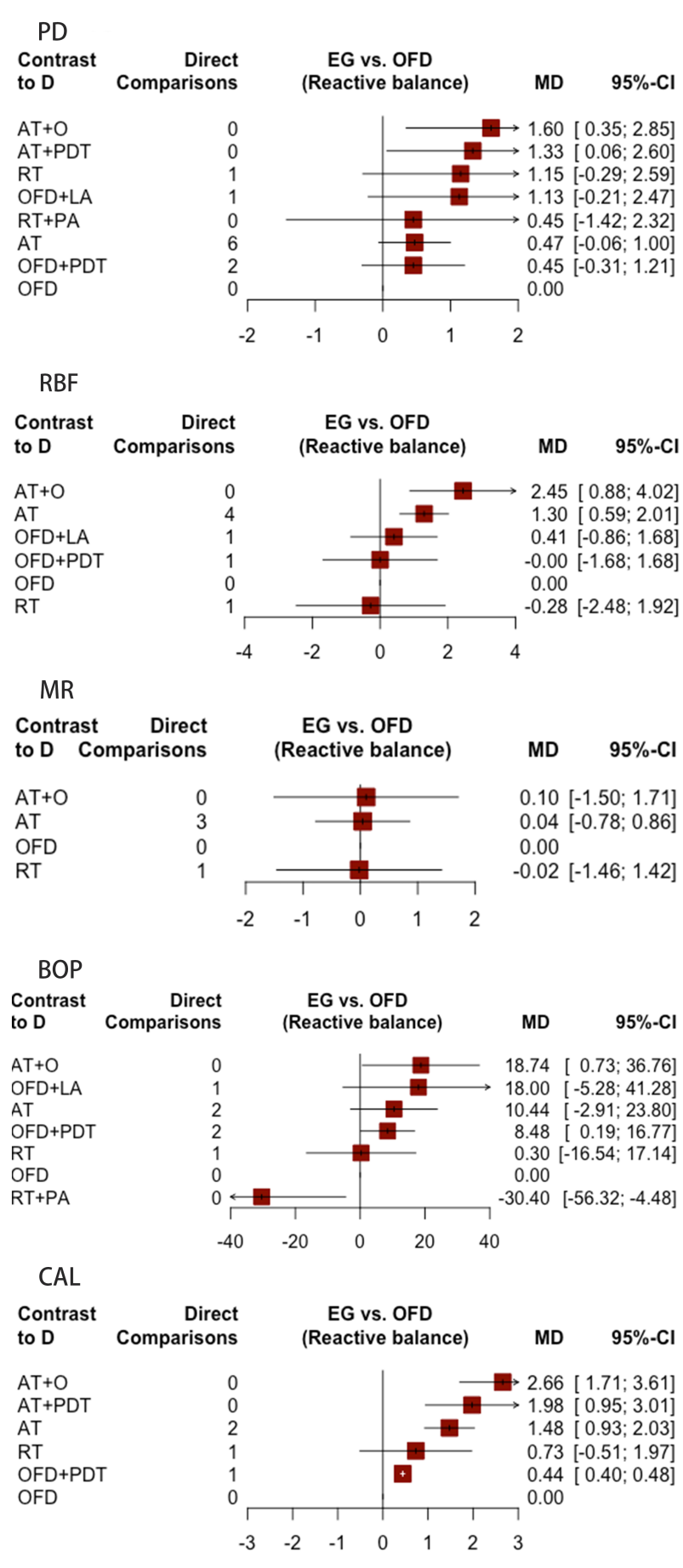


**Figure S4.** pairwise meta-analysis forest plot: other vs ‘OFD’in term of PD

**Appendix 10: League table**

The surgical methods were ranked according to the SUCRA value. The results of NMA are at the bottom left of the table, and the results of a direct comparison are at the top right of the table. While reading the results of the two comparisons, you should read from left to right, and the values between the same column and row are the results of the comparison. Numbers outside of brackets indicate mean difference (MD), and numbers inside of brackets are 95% confidence intervals. If MDs is more than 0, column treatment is better. Bold indicates significant results.

| **AT+O** | / | / | / | / | 1.13 (-0.01, 2.27) | / | / |
| --- | --- | --- | --- | --- | --- | --- | --- |
| 0.27 (-1.35, 1.89) | **AT+PDT** | / | / | / | 0.86 (-0.29, 2.01) | / | / |
| 0.45 (-1.46, 2.36) | 0.18 (-1.74, 2.10) | **RT** | / | 0.70 (-0.49, 1.89) | / | / | 1.15 (-0.29, 2.59) |
| 0.47 (-1.37, 2.31) | 0.20 (-1.65, 2.05) | 0.02 (-1.95, 1.99) | **OFD+LA** | / | / | / | 1.13 (-0.21, 2.47) |
| 1.15 (-1.11, 3.40) | 0.88 (-1.38, 3.14) | 0.70 (-0.49, 1.89) | 0.68 (-1.63, 2.99) | **RT+PA** | / | / | / |
| 1.13 (-0.01, 2.27) | 0.86 (-0.29, 2.01) | 0.68 (-0.86, 2.22) | 0.66 (-0.78, 2.10) | -0.02 (-1.97, 1.93) | **AT** | / | 0.47 (-0.06, 1.00) |
| 1.15 (-0.32, 2.61) | 0.88 (-0.60, 2.36) | 0.70 (-0.93, 2.33) | 0.68 (-0.86, 2.22) | -0.00 (-2.02, 2.02) | 0.02 (-0.90, 0.94) | **OFD+PDT** | 0.45 (-0.31, 1.21) |
| **1.60 (0.35, 2.85)** | **1.33 (0.06, 2.60)** | 1.15 (-0.29, 2.59) | 1.13 (-0.21, 2.47) | 0.45 (-1.42, 2.32) | 0.47 (-0.06, 1.00) | 0.45 (-0.31, 1.21) | **OFD** |

**Table S4.** League table for change in PD reduction (mm) related to surgical treatment.

| **AT+O** | 1.15 (-0.25, 2.55) | / | / | / | / |
| --- | --- | --- | --- | --- | --- |
| 1.15 (-0.25; 2.55) | **AT** | / | / | 1.30 (0.59, 2.01) | / |
| **2.04 (0.02, 4.06)** | 0.89 (-0.57, 2.35) | **OFD+LA** | / | 0.41 (-0.86, 1.68) | / |
| **2.45 (0.15, 4.75)** | 1.30 (-0.53, 3.13) | 0.41 (-1.70, 2.52) | **OFD+PDT** | 0.00 (-1.68, 1.68) | / |
| **2.45 (0.88, 4.02)** | **1.30 (0.59, 2.01)** | 0.41 (-0.86, 1.68) | 0.00 (-1.68, 1.68) | **OFD** | 0.28 (-1.92, 2.48) |
| **2.73 (0.03, 5.43)** | 1.58 (-0.73, 3.89) | 0.69 (-1.85, 3.23) | 0.28 (-2.49, 3.05) | 0.28 (-1.92, 2.48) | **RT** |

**Table S5.**  League table for change in RBF gain (mm) related to surgical treatment.

| **AT+O** | 0.06 (-1.32, 1.44) | / | / |
| --- | --- | --- | --- |
| 0.06 (-1.32, 1.44) | **AT** | 0.04 (-0.78, 0.86) | / |
| 0.10 (-1.50, 1.71) | 0.04 (-0.78, 0.86) | **OFD** | 0.02 (-1.42, 1.46) |
| 0.12 (-2.03, 2.27) | 0.06 (-1.59, 1.71) | 0.02 (-1.42, 1.46) | **RT** |

**Table S6.** League table for change in MR (mm) related to surgical treatment.

| **AT+O** | / | 8.30 ( -3.79, 20.39) | / | / | / | / |
| --- | --- | --- | --- | --- | --- | --- |
| 0.74 (-28.70, 30.18) | **OFD+LA** | / | / | / | 18.00 ( -5.28, 41.28) | / |
| 8.30 ( -3.79, 20.39) | 7.56 (-19.28, 34.40) | **AT** | / | / | 10.44 ( -2.91, 23.80) | **30.70 (11.00, 50.40)** |
| 10.27 ( -9.57, 30.10) | 9.52 (-15.19, 34.24) | 1.97 (-13.75, 17.68) | **OFD+PDT** | / | **8.48 (0.19, 16.77)** | / |
| 18.44 ( -6.22, 43.11) | 17.70 (-11.04, 46.44) | 10.14 (-11.35, 31.64) | 8.18 (-10.60, 26.95) | **RT** | 0.30 (-16.54, 17.14) | / |
| **18.74 (0.73, 36.76)** | 18.00 ( -5.28, 41.28) | 10.44 ( -2.91, 23.80) | **8.48 (0.19, 16.77)** | 0.30 (-16.54, 17.14) | **OFD** | / |
| **39.00 (15.88, 62.12)** | **38.26 (4.96, 71.55)** | **30.70 (11.00, 50.40)** | **28.73 (3.53, 53.94)** | 20.56 ( -8.60, 49.72) | 20.26 ( -3.54, 44.06) | **RT+PA** |

**Table S7.** League table for change in BOP reduction (%) related to surgical treatment.

| **AT+O** | / | 1.18 (0.40, 1.96) | / | / | / |
| --- | --- | --- | --- | --- | --- |
| 0.68 (-0.49, 1.85) | **AT+PDT** | 0.50 (-0.37, 1.37) | / | / | / |
| **1.18 (0.40, 1.96)** | 0.50 (-0.37, 1.37) | **AT** | / | / | **1.48 (0.93, 2.03)** |
| **1.93 (0.37, 3.49)** | 1.25 (-0.36, 2.86) | 0.75 (-0.61, 2.10) | **RT** | / | 0.73 (-0.51, 1.97) |
| **2.22 (1.26, 3.17)** | **1.54 (0.51, 2.57)** | **1.04 (0.49, 1.59)** | 0.29 (-0.95, 1.53) | **OFD+PDT** | **0.44 (0.40, 0.48)** |
| **2.66 (1.71, 3.61)** | **1.98 (0.95, 3.01)** | **1.48 (0.93, 2.03)** | 0.73 (-0.51, 1.97) | **0.44 (0.40, 0.48)** | **OFD** |

**Table S8.** League table for change in CAL gain (mm) related to surgical treatment.

**Appendix 11: SUCRA ranking of all approach**

| **Treatment** | **PD** | | | **RBF** | | | **MR** | | | **BOP** | | | **CAL** | | |
| --- | --- | --- | --- | --- | --- | --- | --- | --- | --- | --- | --- | --- | --- | --- | --- |
|  | **SUCRA** | **Probability of Best (%)** | **Mean Rank** | **SUCRA** | **Probability of Best (%)** | **Mean Rank** | **SUCRA** | **Probability of Best (%)** | **Mean Rank** | **SUCRA** | **Probability of Best (%)** | **Mean Rank** | **SUCRA** | **Probability of Best (%)** | **Mean Rank** |
| OFD | 9.0 | 0.0 | 7.4 | 26.9 | 0.0 | 4.7 | 47.2 | 15.1 | 2.6 | 26.6 | 0.0 | 5.4 | 2.4 | 0.0 | 5.9 |
| AT | 35.1 | 0.0 | 5.5 | 75.3 | 3.6 | 2.2 | 52.6 | 18.1 | 2.4 | 63.0 | 1.5 | 3.2 | 59.7 | 0.0 | 3.0 |
| OFD+LA | 64.7 | 16.9 | 3.5 | 44.8 | 1.3 | 3.8 |  |  |  | 80.1 | 45.7 | 2.2 |  |  |  |
| AT+O | 81.7 | 39.9 | 2.3 | 97.9 | 91.6 | 1.1 | 54.4 | 37.1 | 2.4 | 87.8 | 49.0 | 1.7 | 97.3 | 87.0 | 1.1 |
| OFD+PDT | 34.6 | 0.4 | 5.6 | 30.2 | 1.4 | 4.5 |  |  |  | 57.7 | 2.5 | 3.5 | 26.4 | 0.0 | 4.7 |
| RT | 66.9 | 16.8 | 3.3 | 25.0 | 2.1 | 4.7 | 45.8 | 29.7 | 2.6 | 34.6 | 1.2 | 4.9 | 35.5 | 0.5 | 4.2 |
| RT+PA | 36.1 | 4.3 | 5.5 |  |  |  |  |  |  | 0.3 | 0.0 | 7.0 |  |  |  |
| AT+PDT | 71.9 | 21.8 | 3.0 |  |  |  |  |  |  |  |  |  | 78.7 | 12.5 | 2.1 |

**Table S9.** SUCRA ranking table for surgical treatments of peri-implantitis


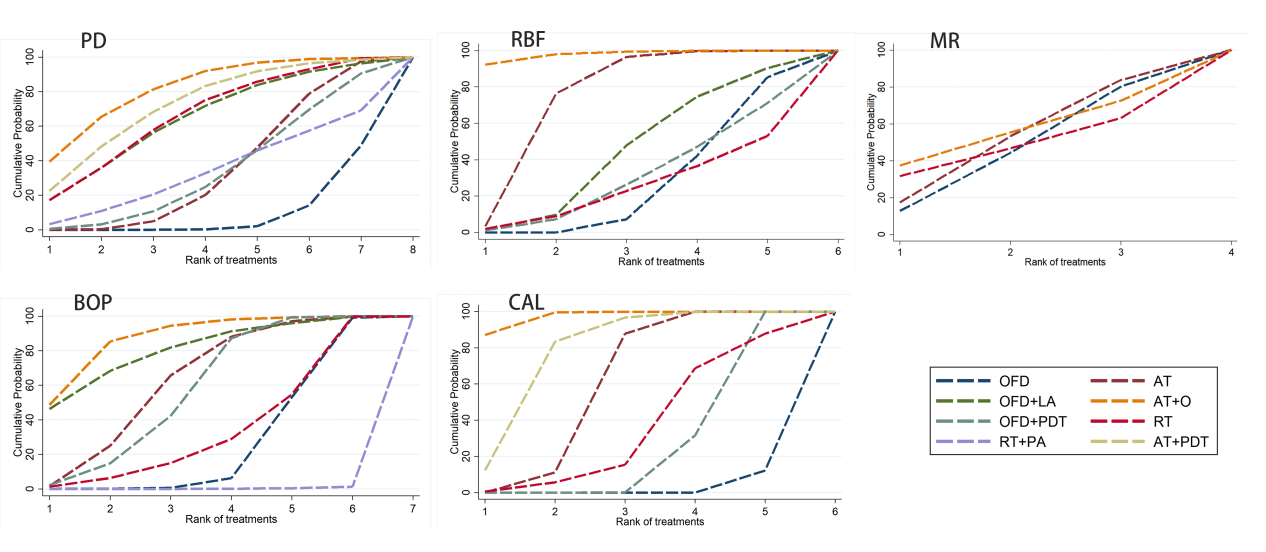


**Figure S5.** Cumulative ranking curves for all five outcomes.

**Appendix 12: Risk of Bias**


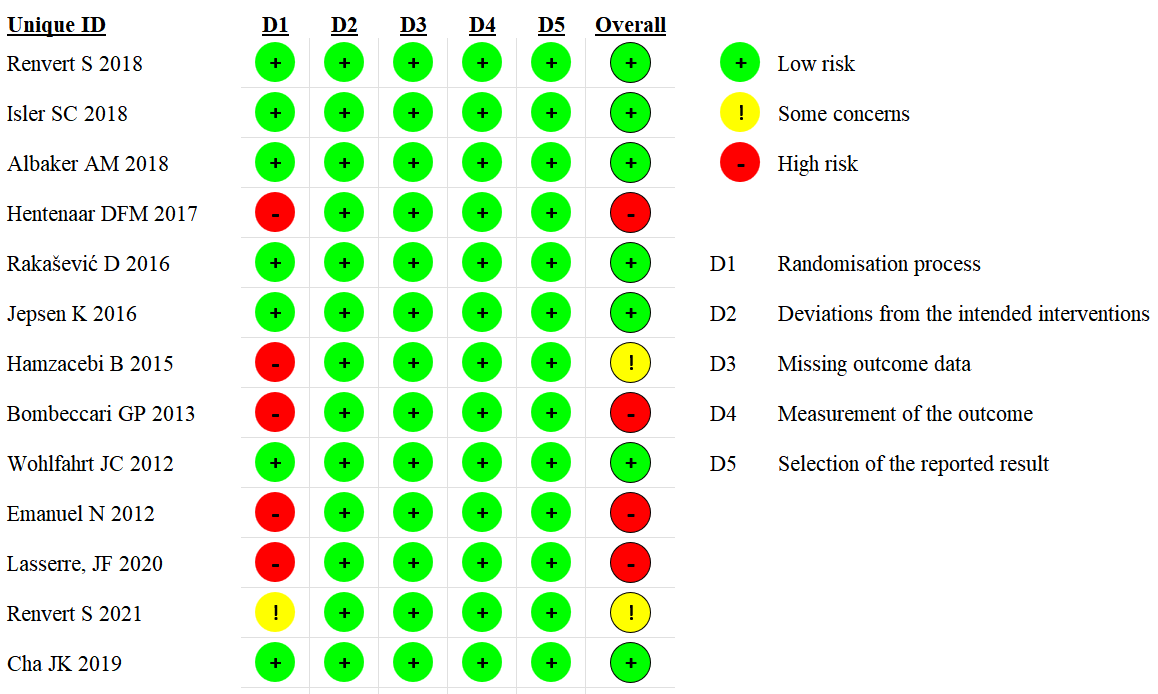


**Figure S6.** Summary of risk of bias: review authors' judgements about each risk of bias item for each included study.


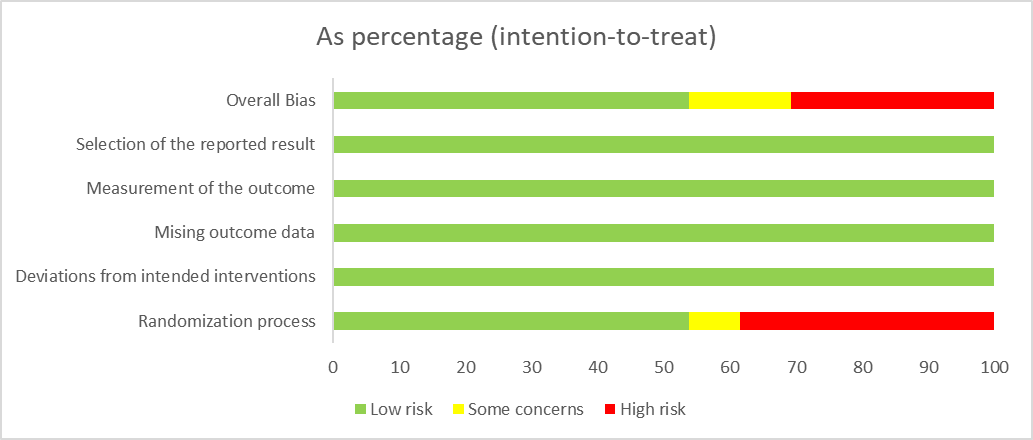


**Figure S7.** Risk of bias summary: review authors' judgements about each risk of bias item for each included study.

| **Unique ID** | **Experimental** | **Comparator** | **Outcome** | **Randomization process** | **Deviations from intended interventions** | **Missing outcome data** | **Measurement of the outcome** | **Selection of the reported result** | **Overall Bias** |
| --- | --- | --- | --- | --- | --- | --- | --- | --- | --- |
| Renvert S 2018 | AT | OFD | PD | Low | Low | Low | Low | Low | Low |
|  |  |  | RBF | Low | Low | Low | Low | Low | Low |
| Isler SC 2018 | AT+O | AT | PD | Low | Low | Low | Low | Low | Low |
|  |  |  | MR | Low | Low | Low | Low | Low | Low |
|  |  |  | BOP | Low | Low | Low | Low | Low | Low |
|  |  |  | CAL | Low | Low | Low | Low | Low | Low |
| Albaker AM 2018 | OFD+PDT | OFD | PD | Low | Low | Low | Low | Low | Low |
|  |  |  | BOP | Low | Low | Low | Low | Low | Low |
| Hentenaar DFM 2017 | RT+PA | RT | PD | High | Low | Low | Low | Low | High |
|  |  |  | BOP | High | Low | Low | Low | Low | High |
| Rakašević D 2016 | AT+PDT | AT | PD | Low | Low | Low | Low | Low | Low |
|  |  |  | CAL | Low | Low | Low | Low | Low | Low |
| Jepsen K 2016 | AT | OFD | PD | Low | Low | Low | Low | Low | Low |
|  |  |  | BOP | Low | Low | Low | Low | Low | Low |
| Hamzacebi B 2015 | AT+PDT | AT | PD | High | Low | Low | Low | Low | Some concerns |
|  |  |  | MR | High | Low | Low | Low | Low | Some concerns |
|  |  |  | BOP | High | Low | Low | Low | Low | Some concerns |
| Bombeccari GP 2013 | OFD+PDT | OFD | PD | High | Low | Low | Low | Low | High |
|  |  |  | BOP | High | Low | Low | Low | Low | High |
|  |  |  | CAL | High | Low | Low | Low | Low | High |
| Wohlfahrt JC 2012 | RT | OFD | PD | Low | Low | Low | Low | Low | Low |
|  |  |  | RBF | Low | Low | Low | Low | Low | Low |
| Emanuel N 2012 | RT | OFD | PD | High | Low | Low | Low | Low | High |
|  |  |  | RBF | High | Low | Low | Low | Low | High |
|  |  |  | MR | High | Low | Low | Low | Low | High |
|  |  |  | CAL | High | Low | Low | Low | Low | High |
| Lasserre JF 2020 | RT | OFD | PD | High | Low | Low | Low | Low | High |
|  |  |  | RBF | High | Low | Low | Low | Low | High |
|  |  |  | MR | High | Low | Low | Low | Low | High |
|  |  |  | BOP | High | Low | Low | Low | Low | High |
|  |  |  | CAL | High | Low | Low | Low | Low | High |
| Renvert S 2021 | AT | OFD | PD | Some concerns | Low | Low | Low | Low | Some concerns |
|  |  |  | RBF | Some concerns | Low | Low | Low | Low | Some concerns |
| Cha JK 2019 | OFD+AT | OFD | PD | Low | Low | Low | Low | Low | Low |
|  |  |  | RBF | Low | Low | Low | Low | Low | Low |
|  |  |  | BOP | Low | Low | Low | Low | Low | Low |

**Table S10.** Bias assessment of 13 RCTs included in main analysis

**Appendix 13: Grading the evidence of the network meta-analysis using CINeMA**

Our judgment given the recommendations of CINeMA online documentation (http://cinema.ispm.ch/#doc) scored all surgical methods. In the following four tables, we listed the evidence level of pairwise comparison of all surgical methods based on CINeMA (Confidence in Network Meta-Analysis), Confidence level: high moderate, low, very low. Downgrading: CINeMA items responsible for downgrading. The quality of evidence for all comparisons was very low, which was caused by within-study bias, imprecision, heterogeneity and incoherence

| Outcome | Comparison | Number of studies | Within-study bias | Reporting bias | Indirectness | Imprecision | Heterogeneity | Incoherence | Confidence rating |
| --- | --- | --- | --- | --- | --- | --- | --- | --- | --- |
| PD | AT: OFD | 6 | Major concerns | Low risk | No concerns | Some concerns | Some concerns | Major concerns | Very low |
|  | OFD: OFD+LA | 1 | Major concerns | Low risk | No concerns | Some concerns | Some concerns | Major concerns | Very low |
|  | OFD: OFD+PDT | 2 | Some concerns | Low risk | No concerns | Some concerns | Some concerns | Major concerns | Very low |
|  | OFD: RT | 1 | No concerns | Low risk | No concerns | Some concerns | Some concerns | Major concerns | Very low |
|  | AT+O: OFD | 0 | Some concerns | Low risk | No concerns | No concerns | Some concerns | Major concerns | Very low |
|  | AT+PDT: OFD | 0 | Major concerns | Low risk | No concerns | No concerns | Some concerns | Major concerns | Very low |
|  | OFD: RT+PA | 0 | Some concerns | Low risk | No concerns | Major concerns | No concerns | Major concerns | Very low |
| RBF | AT: OFD | 4 | Some concerns | Low risk | No concerns | No concerns | Major concerns | Major concerns | Very low |
|  | OFD: OFD +PDT | 1 | No concerns | Low risk | No concerns | Major concerns | No concerns | Major concerns | Very low |
|  | OFD: OFD+LA | 1 | Major concerns | Low risk | No concerns | Major concerns | No concerns | Major concerns | Very low |
|  | OFD: RT | 1 | No concerns | Low risk | No concerns | Major concerns | No concerns | Major concerns | Very low |
|  | AT+O: OFD | 0 | Some concerns | Low risk | No concerns | No concerns | Major concerns | Major concerns | Very low |
| MR | AT: OFD | 3 | Major concerns | Low risk | No concerns | Some concerns | Some concerns | Major concerns | Very low |
|  | OFD: RT | 1 | No concerns | Low risk | No concerns | Major concerns | No concerns | Major concerns | Very low |
|  | AT+O: OFD | 0 | Some concerns | Low risk | No concerns | Major concerns | No concerns | Major concerns | Very low |
| BOP | AT: OFD | 2 | Major concerns | Low risk | No concerns | Major concerns | No concerns | Major concerns | Very low |
|  | OFD: OFD+LA | 1 | Major concerns | Low risk | No concerns | Major concerns | No concerns | Major concerns | Very low |
|  | OFD: OFD+PDT | 2 | No concerns | Low risk | No concerns | No concerns | Major concerns | Major concerns | Very low |
|  | OFD: RT | 1 | No concerns | Low risk | No concerns | Major concerns | No concerns | Major concerns | Very low |
|  | AT+O: OFD | 0 | Major concerns | Low risk | No concerns | No concerns | Major concerns | Major concerns | Very low |
|  | OFD:RT+PA | 0 | Some concerns | Low risk | No concerns | No concerns | Major concerns | Major concerns | Very low |
| CAL | AT: OFD | 2 | Major concerns | Low risk | No concerns | Major concerns | Major concerns | Major concerns | Very low |
|  | OFD: OFD+PDT | 1 | Some concerns | Low risk | No concerns | Major concerns | Major concerns | Major concerns | Very low |
|  | OFD: RT | 1 | No concerns | Low risk | No concerns | Some concerns | Some concerns | Major concerns | Very low |
|  | AT+O: OFD | 0 | Some concerns | Low risk | No concerns | Major concerns | Major concerns | Major concerns | Very low |
|  | AT+PDT: OFD | 0 | Major concerns | Low risk | No concerns | No  concerns | No  concerns | Major concerns | Very low |

**Table S11. CINeMA confidence rating for five outcomes**
